# Time- and Race-Specific Haematological Reference Intervals for Healthy Volunteer Trials: A Retrospective Analysis of Pooled Data From Multiple Phase I Trials

**DOI:** 10.3389/fphar.2020.00314

**Published:** 2020-03-13

**Authors:** Simon Coates, Duolao Wang, Tomasz Pierscionek, Sara Fernandes, Dilshat Djumanov, Ulrike Lorch, Jörg Täubel

**Affiliations:** ^1^ Richmond Pharmacology, London, United Kingdom; ^2^ Department of Clinical Sciences, Liverpool School of Tropical Medicine, Liverpool, United Kingdom; ^3^ Cardiovascular and Cell Sciences Research Institute, St George’s, University of London, London, United Kingdom

**Keywords:** white blood cell, racial, diurnal, inclusion criteria, reference intervals

## Abstract

Most UK hospitals, laboratories, and research institutions use uniform reference intervals (RI) that do not take into account known diurnal and racial variation in total white blood cells (WBC) count and its constituent parameters. These risks of excluding potentially suitable ethnic minority volunteers from participating in phase I clinical trials could call into question the validity of a trial’s findings or limit its scientific applications and ability to accurately observe drug effects upon WBC parameters. This study pools data from multiple phase I trials, assesses the effects of race and time of day on WBC count, and compares it to the existing literature to establish race and time-specific RIs. A total 13,332 venous blood samples obtained from 7,157 healthy male and female volunteers at the time of screening or admission (predosing) who took part in 35 phase I trials over a period of seven years were pooled and the data were analyzed using generalised estimating equation models. Adjusted RI of total WBC count and its individual parameters were then calculated according to time of day (morning vs. evening) for both black and nonblack populations. This study indicates that black individuals on average had lower total WBC, neutrophil, monocyte, eosinophil, and basophil counts than individuals from nonblack racial groups. Black volunteers had higher mean lymphocyte counts relative to their nonblack counterparts. These differences were deemed statistically significant. Statistically significant increases in total WBC, neutrophil, lymphocyte, and monocyte counts were also observed over the course of daily sampling. Eosinophil counts decreased during this time period, but this finding was only statistically significant in the nonblack population. Despite an observed mild diurnal increase in basophil count in both populations, this was not considered statistically significant. This high-powered study adds significant weight to the known evidence for diurnal and racial variation in WBC parameters. Importantly, it proposes specific RIs that more precisely reflect race and time of day. These could ensure increased participation of black volunteers in clinical trials for improved population representation. Furthermore, the proposed RIs allow for more accurate postdose safety monitoring and reporting, and ensure improved monitoring of postdose WBC count changes.

## Introduction

Most UK hospitals, laboratories, CROs, and other research institutions use uniform reference intervals (RI) for white blood cell (WBC) parameters, irrespective of an individual’s race, gender, age, or the time of day. However, as described below, genetic and diurnal variation in WBC parameters is an acknowledged phenomenon. Adopting a single reference range is thus potentially unsuitable and could result in the underrepresentation of volunteers from ethnic minority groups in early phase clinical trials (Haley et al., 2017; Sheffet et al., 2018). Furthermore, a poor representation of these groups in phase I studies may call into question the validity of a clinical trial’s findings (Hussain-Gambles et al., 2004), limit its scientific applications, and amplify health disparities (Chen et al., 2014). Low participation rates in clinical trials by historically underrepresented groups has previously contributed to failures in detecting potential harm as population representation proved insufficient to spot negative effects (Roberts et al., 2012). A single reference range may also limit the ability to accurately observe drug effects on WBC parameters.

A single WBC reference range does not take account of genetically determined lower neutrophil counts reported in subjects of African ancestry (Nalls et al., 2008; Reich et al., 2009; Thobakgale and Ndung'u, 2014). These individuals have lower neutrophil and total WBC counts and are at high risk of being barred from participation in phase I studies. An absolute neutrophil count (ANC) of 1.5 × 10^9^ cells/liter is often deemed the minimum threshold for an individual to be included in a clinical trial. Individuals with a WBC <1.5 × 10^9^ cells/liter that is not secondary to any pathology and who are otherwise healthy are deemed to have Benign Ethnic Neutropenia (BEN) (Hsieh et al., 2005), yet are frequently excluded from trial participation. BEN occurs in <1% of individuals of European ancestry (Hsieh, 2007), yet has a prevalence of up to 50% in individuals of African descent (Haddy et al., 1999; Keller et al., 2014; Tandeter et al., 2016; Rahmani et al., 2017) because of the near 100% prevalence of the Duffy Antigen Receptor for Chemokines (DARC) allele (rs2814778) found across sub-Saharan populations (McManus et al., 2017) and associated with lower neutrophil counts (Hsieh et al., 2007; Crosslin et al., 2012).

The scientific literature highlights that both total WBCs and constituent cells (neutrophils, monocytes, lymphocytes, basophils, eosinophils) vary across the diurnal cycle (Doan and Zefas, 1927; McKee et al., 2011; Sennels et al., 2011; Ackermann et al., 2012; Ella et al., 2016; Souto-Filho et al., 2019), with neutrophils peaking in the evening and being at their lowest in the morning (Sennels et al., 2011; Ella et al., 2016). Furthermore, neutrophil and monocyte migration from blood to tissue showed circadian oscillations that cause variations in the magnitude of inflammatory responses (Scheiermann et al., 2012; Scheiermann et al., 2013) and diurnal variation of lymphocyte trafficking appears to be under adrenergic control (Suzuki et al., 2016). This is a significant observation as blood samples are typically obtained at various time points during a trial and there is a risk that diurnal variation in WBC could be wrongly attributed to the activity of an Investigational Medicinal Product (IMP)—defined as “an active ingredient or placebo that has been pharmaceutically formulated (prepared) for human use which is being tested, or used as a comparator, in a clinical trial” (EUPATI, 2019).

Single RIs for each WBC parameter that fail to consider racial and diurnal variation, arguably, lack validity and could result in clinicians being misled about any potential drug effects and the status of an individual’s immune system, resulting in their inappropriate exclusion from a clinical trial. This study pools WBC parameters from multiple phase I trials, assesses the effects of race and time of day on WBC count, and compares these to the existing literature. This data was then used to propose specific RIs that more precisely reflect the ethnic variation seen in a diverse population of volunteers and the time of day a sample was taken.

## Methods

### Study Population

This study analyzed pooled data from 35 clinical trials conducted over a period of seven years (January 2010–January 2017) at Richmond Pharmacology, St George’s, University of London, UK. All subjects provided written informed consent for that specific clinical trial and all 35 trials were approved by the Medicines and Healthcare Products Regulatory Agency (MHRA) and a Research Ethics Committee. Additionally, all 35 trials were performed in accordance with guidelines established by the Declaration of Helsinki and the International Conference on Harmonisation Good Clinical Practice guidelines.

WBC count results from 13,332 venous blood samples obtained from 7,157 healthy subjects (2,668 women and 4,489 men) who had taken part in one or more of these 35 studies were obtained from Richmond Pharmacology’s clinical trials database. The anonymized WBC count data from these samples and used in this research was obtained in accordance with the Health Research Authority and the Medical Research Council’s advice on the use of anonymized data (Medical Research Council: data and tissues tool kit; Health Research Authority defining research table 2017; Health Research Authority decision tools).

All subjects were aged 18 to 76 years, nonsmokers, had not consumed caffeine over the preceding 48 h, and their urine tested negative for alcohol consumption and for Drugs of Abuse (DOA). These criteria were checked for consistency with inclusion criteria pertaining to the individual studies from which the data was obtained.

The haematological results derived from samples obtained during a screening visit prior to inclusion in a study or at the time of admission to the clinical trial unit prior to exposure to any study related medication (samples obtained during the screening process, on Day -2, Day -1, or Day 1 predose). Consequently, the results are not confounded by the effects of any IMP or non-IMP.

### Number of Subjects, Blood Samples, and Parameters

The 7,157 individual subjects whose data were used in this study provided 13,332 blood samples. From this, a total of 76,668 individual blood parameters were available for the analysis of the following parameters: total WBC count, neutrophils, lymphocytes, monocytes, basophils, and eosinophils. **Table 1** shows the number of blood samples obtained per subject. The majority of subjects (5,829) gave only one or two samples; subjects providing more samples typically did so on account of repeat assessments (e.g., at screening or admission) or due to participation in more than one study over the seven-year period. The greatest number of samples obtained was 24 samples from a single subject who had participated in seven clinical trials during the study period.

**Table 1:** Number of blood samples per subject.

Number of Samples	Number of subjects
1	4,138
2	1,691
3	613
4	294
5	164
6	85
7	65
8	31
9	28
10	20
11	8
12	5
13	3
14	4
15	1
16	1
17	2
18	1
21	1
22	1
24	1
**Total**	**7,157**

The use of more than one sample per subject was allowed in the methodology for the following reasons:

- It took into account normal white cell physiology and intrasubject variation.
- It increased the number of unique blood samples available for analysis.
- The larger sample size increases the power of the statistical analyses, i.e., any abnormal results that were captured within the population have a reduced confounding effect.

Where a subject gave repeated blood samples at a single time point, an average was calculated before statistical analyses. This produced 74,074 blood parameters that were used for descriptive and inferential statistical analyses.

### Blood Sampling and Haematological Analysis

Samples were obtained *via* venepuncture using standardized aseptic technique by trained staff throughout the day. The number of samples collected at each time point across the day is shown in **Table 3**. Venous blood samples (4 ml) were obtained from each subject using Vacutainer^®^ CPT™ tubes (Beckton Dickenson, Wokingham, UK). The tubes were placed on a roller mixer until WBC counting took place. To maintain the stability of the samples they were processed further within 60 min. Cell counts were carried out within 5 h after the blood was drawn by an accredited pathology laboratory (The Doctors Laboratory Limited, London, UK). Cells were counted according to The Doctor’s Laboratory’s standard protocols.

Samples were centrifuged at room temperature in a horizontal rotor for 20 min at 1,800 g. Mononuclear cells were isolated from the whitish layer under the plasma layer, while neutrophils were separated from the layer above the erythrocytes. Preparations were used when purity obtained by nuclear staining (methylene-blue) was higher than 97%. Isolated cells were immediately processed.

### Statistical Analysis

The data obtained from the haematological investigations of the subjects’ samples were grouped by their self-ascribed race into the following groups: black (black African & black Caribbean) and nonblack (Caucasian and Asian). Their self-ascribed “race” was ascertained at the initial telephone recruitment stage.

Mean values and standard deviations per time of day were calculated for each racial group for all haematological parameters.

The haematological parameters were analyzed by generalised estimating equation (GEE) models in which race, time, and interaction between time and race were treated as fixed effects, age, sex, and fast status as covariates, the subject as a cluster effect. The GEE model has a normal distribution and identity link function with exchangeable covariance structure. The estimated between-race differences at each time point and within-race differences (morning vs. evening) in marginal means from the GEE models are therefore derived and reported together with their 95% confidence intervals. Model parameters shown in **Supplementary Table**.

Reported P-values are two-sided and a P-value of <0.05 was considered statistically significant. Adjusted normal intervals of haematological parameters were calculated for black and nonblack populations and for samples collected in the morning versus samples collected in the evening. All statistical analyses were carried out using the Statistical Analysis System (SAS) version 9.3 (SAS Institute, Inc., Cary, NC, USA).

## Results

### Subject Characteristics

Blood samples collected from 7,157 subjects were analyzed. 926 (12.94%) of these subjects were black and 6,227 (87.06%) were nonblack (**Table 2**). A large proportion (96.1%) of the analyzed samples were obtained from fasted subjects.

**Table 2:** Baseline characteristics of subjects.

Variable	Statistics	Race		All
		Black	Nonblack	
Age	Number of subjects, mean age (Standard Deviation)	926,30.29(10.16)	6227,31.40(11.24)	7157,31.26(11.11)
Sex	N	926	6227	7157
	Female	410(44.3%)	2,258(36.2%)	2,668(37.3%)
	Male	516(55.7%)	3,973(63.8%)	4,489(62.7%)
Fasted	N	926	6,227	7,157
	No	27(2.9%)	249(4.0%)	276(3.9%)
	Yes	899(97.1%)	5,982(96.0%)	6,881(96.1%)

The neutrophil count represented roughly half of the total WBC count while lymphocytes comprised about 35% of the total. Monocytes (≈10%), eosinophils (2%–3%), and basophils (<1%) accounted for the remainder of the WBC numbers.

### Diurnal Differences in Blood Cell Counts From 8 am to 6 pm

**Figure 1:**
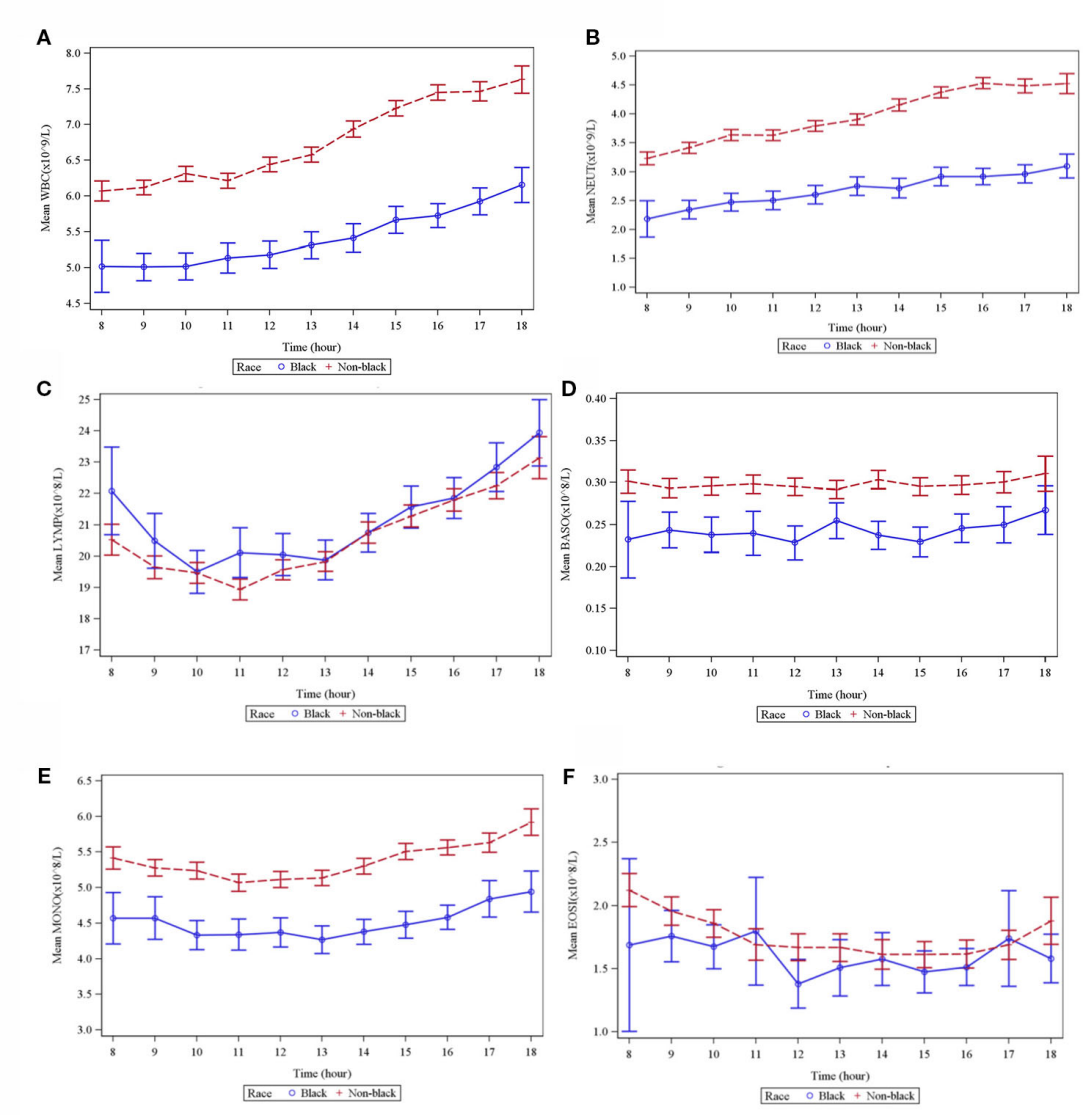
Graphs showing means of different WBC types by time and race. **(A)** Total WBC. **(B)** Neutrophils. **(C)** Lymphocytes. **(D)** Basophils. **(E)** Monocytes. **(F)** Eosinophils.

Diurnal variation in the total WBC and its constituent parameters was measured at different time points for each race group and for the total sample population (**Table 3**). Overall, the combined mean WBC count for both populations (black and nonblack) was 24% (1.40 × 10^9^ cells/liter) higher at the end of the daily sampling period (6 pm) relative to the morning (8 am). For black individuals, total WBC count rose by an average of 20% from the morning until the end of daily sampling, while the increase for nonblack volunteers was 29% (**Figure 1A**). A substantial proportion of this rise can be attributed to an overall 36% (1.08 × 10^9^ cells/liter) increase in the combined mean neutrophil count during the course of daily sampling, a 34% rise for black and 44% rise for nonblack volunteers, respectively (**Figure 1B**).

**Table 3:** Summary statistics of haematological parameters [Basophils, Eosinophils, Neutrophils, Lymphocytes, Monocytes, and white blood cell (WBC)] for black and nonblack populations by time of day.

Parameter (x10^8^/liter)	Hour	Number of subjects, mean number of cells (Standard Deviation)		
		Black	Nonblack	All
Basophils	8	30, 0.21(0.13)	426, 0.30(0.18)	456, 0.29(0.18)
	9	96, 0.22(0.12)	879, 0.29(0.19)	975, 0.29(0.18)
	10	141, 0.23(0.16)	1,113, 0.30(0.19)	1,254, 0.30(0.19)
	11	141, 0.27(0.22)	1,006, 0.30(0.17)	1,147, 0.29(0.18)
	12	206, 0.23(0.19)	1,442, 0.29(0.17)	1,648, 0.29(0.18)
	13	191, 0.25(0.15)	1,394, 0.29(0.19)	1,585, 0.28(0.19)
	14	192, 0.25(0.15)	1,165, 0.29(0.18)	1,357, 0.28(0.17)
	15	208, 0.23(0.15)	1,297, 0.29(0.18)	1,505, 0.28(0.17)
	16	294, 0.24(0.14)	1,334, 0.29(0.17)	1,628, 0.28(0.17)
	17	144, 0.24(0.14)	660, 0.29(0.16)	804, 0.28(0.16)
	18	85, 0.26(0.19)	270, 0.31(0.19)	355, 0.30(0.19)
Eosinophils	8	30, 2.10(1.90)	426, 2.08(1.97)	456, 2.08(1.96)
	9	96, 2.21(2.66)	879, 1.81(1.35)	975, 1.85(1.53)
	10	141, 1.71(2.03)	1,114, 1.87(1.89)	1255, 1.85(1.90)
	11	141, 2.03(3.16)	1,006, 1.57(1.87)	1147, 1.62(2.08)
	12	207, 1.58(2.06)	1,443, 1.56(1.54)	1650, 1.57(1.62)
	13	191, 1.47(1.80)	1,398, 1.49(1.48)	1589, 1.49(1.52)
	14	192, 1.49(1.77)	1,165, 1.52(1.50)	1357, 1.52(1.54)
	15	208, 1.31(1.15)	1,300, 1.55(1.55)	1508, 1.51(1.51)
	16	294, 1.33(1.29)	1,337, 1.60(1.47)	1631, 1.55(1.44)
	17	145, 1.74(2.36)	664, 1.65(1.61)	809, 1.67(1.77)
	18	85, 1.38(1.18)	272, 1.67(1.44)	357, 1.60(1.38)
Lymphocytes	8	31, 21.55(5.06)	426, 19.78(5.83)	457, 19.90(5.79)
	9	96, 19.88(5.06)	880, 18.68(5.14)	976, 18.80(5.14)
	10	142, 18.86(5.26)	1,114, 19.20(5.31)	1256, 19.16(5.30)
	11	141, 19.53(5.90)	1,010, 18.53(5.04)	1151, 18.65(5.16)
	12	206, 19.31(5.74)	1,444, 19.07(5.28)	1650, 19.10(5.33)
	13	191, 19.57(4.91)	1,394, 19.41(5.26)	1585, 19.43(5.22)
	14	192, 20.24(5.08)	1,166, 20.21(5.60)	1358, 20.22(5.53)
	15	209, 20.81(5.98)	1,299, 21.00(6.01)	1508, 20.97(6.01)
	16	296, 21.57(6.29)	1,338, 21.60(6.06)	1634, 21.60(6.10)
	17	145, 21.85(5.51)	665, 21.76(6.01)	810, 21.78(5.92)
	18	86, 23.46(6.08)	273, 22.96(6.82)	359, 23.08(6.65)
Monocytes	8	30, 4.37(1.16)	426, 5.13(1.74)	456, 5.08(1.72)
	9	96, 4.62(1.85)	879, 5.08(1.72)	975, 5.04(1.74)
	10	141, 4.23(1.51)	1,113, 5.23(1.81)	1,254, 5.12(1.81)
	11	141, 4.35(1.67)	1,006, 4.94(1.68)	1,147, 4.87(1.69)
	12	206, 4.21(1.61)	1,443, 4.98(1.70)	1,649, 4.89(1.71)
	13	191, 4.33(1.37)	1,394, 5.05(1.65)	1,585, 4.96(1.64)
	14	192, 4.17(1.35)	1,165, 5.16(1.62)	1,357, 5.02(1.62)
	15	208, 4.29(1.39)	1,297, 5.44(1.70)	1,505, 5.28(1.71)
	16	294, 4.45(1.44)	1,335, 5.48(1.62)	1,629, 5.30(1.64)
	17	145, 4.73(1.61)	660, 5.54(1.68)	805, 5.40(1.69)
	18	85, 4.79(1.57)	271, 5.77(1.67)	356, 5.54(1.70)
Neutrophils (x10^9^/liter)	8	31, 2.36(0.96)	427, 3.07(1.11)	458, 3.02(1.11)
	9	97, 2.34(1.00)	883, 3.30(1.20)	980, 3.20(1.22)
	10	143, 2.49(0.96)	1,115, 3.55(1.30)	1,258, 3.43(1.31)
	11	143, 2.45(1.11)	1,009, 3.56(1.26)	1,152, 3.42(1.30)
	12	206, 2.60(1.28)	1,442, 3.73(1.38)	1,648, 3.59(1.42)
	13	193, 2.78(1.19)	1,399, 3.87(1.46)	1,592, 3.74(1.47)
	14	198, 2.69(1.28)	1,166, 4.09(1.50)	1,364, 3.89(1.55)
	15	209, 2.89(1.26)	1,300, 4.34(1.48)	1,509, 4.14(1.53)
	16	298, 2.90(1.24)	1,342, 4.49(1.49)	1,640, 4.21(1.57)
	17	154, 2.87(1.11)	673, 4.40(1.52)	827, 4.12(1.57)
	18	97, 3.16(1.38)	283, 4.42(1.64)	380, 4.10(1.67)
White Blood Cells (x10^9^/liter)	8	32, 5.17(1.18)	427, 5.80(1.54)	459, 5.76(1.52)
	9	98, 5.01(1.25)	886, 5.88(1.48)	984, 5.80(1.48)
	10	144, 4.99(1.26)	1,126, 6.21(1.62)	1,270, 6.07(1.63)
	11	147, 5.06(1.51)	1,043, 6.08(1.59)	1,190, 5.96(1.61)
	12	211, 5.14(1.58)	1,477, 6.31(1.68)	1,688, 6.17(1.72)
	13	196, 5.37(1.42)	1,411, 6.49(1.76)	1,607, 6.36(1.76)
	14	198, 5.32(1.51)	1,169, 6.81(1.79)	1,367, 6.60(1.83)
	15	209, 5.55(1.59)	1,306, 7.17(1.80)	1,515, 6.95(1.86)
	16	300, 5.66(1.57)	1,349, 7.40(1.79)	1,649, 7.08(1.88)
	17	155, 5.74(1.34)	681, 7.33(1.81)	836, 7.04(1.84)
	18	97, 6.19(1.71)	288, 7.49(1.92)	385, 7.16(1.95)

The combined mean lymphocyte count also rose by 16% (0.318 × 10^9^ cells/liter)—a 9% diurnal rise for blacks and a 16% rise for nonblack volunteers respectively (**Figure 1C**).

Combined mean basophil and monocyte counts rose 3.4% (0.001 × 10^9^ cells/liter) (a 24% rise in black and a 3% rise in nonblack volunteers) (**Figure 1D**) and 9% (0.046 × 10^9^ cells/liter) (a 9.6% rise in black and a 12.5% rise in nonblack volunteers) (**Figure 1E**) respectively between 8 am and 6 pm. Contrary to other parameters, the combined mean eosinophil count showed a 30% decrease throughout the day (0.048 × 10^9^ cells/liter)—a 52% decrease in black and a 25% decrease in nonblack volunteers respectively (**Figure 1F**).

### Comparison of WBC Parameters in Black and Nonblack Subjects

Total WBC counts from black volunteers were on average lower than those for nonblack volunteers (**Table 4**). Using the GEE model analysis (**Table 4**), relative to nonblack volunteers, black volunteers had a lower total WBC count across all time points (−1.35 × 10^9^ cells/liter, P < 0.0001) (**Table 4**), a lower mean neutrophil count (−1.29 × 10^9^ cells/liter, P < 0.0001) (**Table 4**), a lower mean monocyte count (−0.86 × 10^8^ cells/liter, P < 0.0001) (**Table 4**), a lower mean basophil count (−0.06 × 10^8^ cells/liter, P < 0.0001) (**Table 4**), and a lower mean eosinophil count −0.15 × 10^8^ cells/liter, P = 0.0097) (**Table 4**). However, black subjects had higher mean lymphocyte counts relative to their nonblack counterparts (0.53 × 10^8^ cells/liter, P = 0.0082) (**Table 4**).

**Table 4:** Generalised estimating equation (GEE) model analysis of haematological parameters [Basophils, Eosinophils, Lymphocytes, Monocytes, Neutrophils, and white blood cell (WBC)]: between and within group comparison.

Parameter (x10^8^/liter)	Comparison	Hour	Difference	95% CI		Probability
				Lower Limit	Upper Limit	
Basophils	Blacks vs Nonblacks	8	−0.07	−0.12	−0.02	0.0038
		9	−0.05	−0.07	−0.03	<0.0001
		10	−0.06	−0.08	−0.04	<0.0001
		11	−0.06	−0.08	−0.03	<0.0001
		12	−0.07	−0.09	−0.05	<0.0001
		13	−0.04	−0.06	−0.02	0.0009
		14	−0.07	−0.08	−0.05	<0.0001
		15	−0.07	−0.08	−0.05	<0.0001
		16	−0.05	−0.07	−0.03	<0.0001
		17	−0.05	−0.07	−0.03	<0.0001
		18	−0.04	−0.08	−0.01	0.0127
		All time points	−0.06	−0.07	−0.04	<0.0001
	Morning vs Evening	Blacks	−0.02	−0.04	0.01	0.1352
		Nonblacks	−0.01	−0.01	0.00	0.2083
		All	−0.01	−0.02	0.00	0.0611
Eosinophils	Blacks vs Nonblacks	8	−0.44	−1.17	0.30	0.2430
		9	−0.20	−0.42	0.02	0.0793
		10	−0.19	−0.38	0.01	0.0584
		11	0.11	−0.28	0.49	0.5957
		12	−0.29	−0.51	−0.07	0.0110
		13	−0.16	−0.37	0.05	0.1378
		14	−0.04	−0.24	0.16	0.7168
		15	−0.14	−0.31	0.03	0.1119
		16	−0.10	−0.27	0.06	0.2220
		17	0.05	−0.30	0.40	0.7815
		18	−0.30	−0.55	−0.05	0.0176
		All time points	−0.15	−0.27	−0.04	0.0097
	Morning vs Evening	Blacks	0.09	−0.29	0.48	0.6289
		Nonblacks	0.25	0.17	0.33	<0.0001
		All	0.17	−0.02	0.36	0.0806
Lymphocytes	Blacks vs Nonblacks	8	1.56	0.10	3.02	0.0367
		9	0.85	−0.05	1.74	0.0629
		10	0.04	−0.65	0.72	0.9207
		11	1.18	0.38	1.98	0.0040
		12	0.48	−0.20	1.15	0.1667
		13	0.05	−0.58	0.69	0.8741
		14	−0.01	−0.65	0.64	0.9809
		15	0.29	−0.41	0.98	0.4203
		16	0.06	−0.61	0.73	0.8576
		17	0.60	−0.25	1.44	0.1658
		18	0.80	−0.43	2.03	0.2021
		All time points	0.53	0.14	0.93	0.0082
	Morning vs Evening	Blacks	−2.17	−2.92	−1.41	<0.0001
		Nonblacks	−2.49	−2.82	−2.16	<0.0001
		All	−2.33	−2.74	−1.91	<0.0001
Monocytes	Blacks vs Nonblacks	8	−0.85	−1.23	−0.46	<0.0001
		9	−0.70	−1.00	−0.41	<0.0001
		10	−0.91	−1.11	−0.70	<0.0001
		11	−0.73	−0.95	−0.51	<0.0001
		12	−0.74	−0.95	−0.54	<0.0001
		13	−0.87	−1.06	−0.67	<0.0001
		14	−0.92	−1.10	−0.75	<0.0001
		15	−1.03	−1.22	−0.84	<0.0001
		16	−0.98	−1.15	−0.81	<0.0001
		17	−0.79	−1.06	−0.52	<0.0001
		18	−0.97	−1.30	−0.65	<0.0001
		All time points	−0.86	−0.97	−0.75	<0.0001
	Morning vs evening	Blacks	−0.30	−0.51	−0.08	0.0083
		Nonblacks	−0.39	−0.48	−0.30	<0.0001
		All	−0.34	−0.46	−0.22	<0.0001
Neutrophils (x10^9^/liter)	Blacks vs Nonblacks	8	−1.05	−1.37	−0.72	<0.0001
		9	−1.07	−1.23	−0.91	<0.0001
		10	−1.16	−1.32	−1.00	<0.0001
		11	−1.13	−1.29	−0.97	<0.0001
		12	−1.19	−1.35	−1.03	<0.0001
		13	−1.16	−1.32	−0.99	<0.0001
		14	−1.44	−1.62	−1.26	<0.0001
		15	−1.46	−1.62	−1.29	<0.0001
		16	−1.61	−1.76	−1.47	<0.0001
		17	−1.52	−1.71	−1.34	<0.0001
		18	−1.43	−1.69	−1.16	<0.0001
		All time points	−1.29	−1.38	−1.21	<0.0001
	Morning vs evening	Blacks	−0.65	−0.80	−0.50	<0.0001
		Nonblacks	−1.08	−1.16	−0.99	<0.0001
		All	−0.86	−0.95	−0.78	<0.0001
White Blood Cells (x10^9^/liter)	Blacks vs Nonblacks	8	−1.05	−1.43	−0.67	<0.0001
		9	−1.11	−1.30	−0.91	<0.0001
		10	−1.29	−1.48	−1.10	<0.0001
		11	−1.08	−1.29	−0.87	<0.0001
		12	−1.26	−1.46	−1.07	<0.0001
		13	−1.27	−1.46	−1.07	<0.0001
		14	−1.52	−1.73	−1.32	<0.0001
		15	−1.56	−1.76	−1.36	<0.0001
		16	−1.73	−1.91	−1.55	<0.0001
		17	−1.54	−1.76	−1.32	<0.0001
		18	−1.48	−1.78	−1.17	<0.0001
		All time points	−1.35	−1.46	−1.25	<0.0001
	Morning vs evening	Blacks	−0.91	−1.08	−0.73	<0.0001
		Nonblacks	−1.34	−1.43	−1.24	<0.0001
		All	−1.12	−1.22	−1.02	<0.0001

An overlap in cell count for both racial groups was observed with respect to lymphocytes (**Figure 1C**) and eosinophils (**Figure 1F**). No overlap was observed in the case of total WBC count (**Figure 1A**), neutrophils (**Figure 1B**), basophils (**Figure 1D**) monocytes (**Figure 1E**).

Comparison of WBC Parameters in Samples Collected in the Morning Versus Samples Collected in the Evening in Black and Nonblack Subjects

WBC counts in black and nonblack subjects increased from 8 am to 6 pm. When data from both groups were pooled, the difference was statistically significant for total WBC counts (−1.12, P < 0.0001), neutrophil counts (−0.86, P < 0.0001), lymphocyte counts (−2.33, P < 0.0001) and monocyte counts (−0.34, P < 0.0001) (**Table 4**). The decrease in eosinophil count during the sampling period was significant only in nonblack subjects (0.25, P < 0.0001) (**Table 4**). Dirunal basophil variation was not significantly different between 8 am and 6 pm for either black (−0.02, P = 0.1352) or nonblack subjects (−0.01, P = 0.2083) or when both groups were combined (−0.01, P = 0.0611).

### Proposed New References Intervals

**Table 5** displays lower and upper normal limits of proposed new RIs based on race (black and nonblack populations) and time of day (morning and evening) for total WBC count and individual parameters. For comparison, the RIs from the lab that analyzed the samples in this study (The Doctors Laboratory) are shown in **Table 6**. **Table 6** also shows the RIs from a range of NHS hospitals, private clinics and haematological charities from across the UK (available in the public domain), which confirms that The Doctor’s Laboratory’s RIs are consistent with those used throughout the UK and are therefore suitable for use as comparative RIs in this manuscript.

**Table 5:** Estimated reference ranges of white blood cell (WBC), neutrophils, lymphocytes, monocytes, eosinophils, and basophils by race and time of day.

Parameter (×10^9^/liter)	Race	Morning		Evening	
		No. of subjects	Reference range	No. of subjects	Reference range
WBC	Black	192	2.7–7.3	437	2.9–8.7
	Nonblack	1,836	3.0–9.1	2005	3.9–10.9
Neutrophils	Black	191	0.6–4.2	434	0.6–5.3
	Nonblack	1,832	1.0–5.8	1993	1.6–7.4
Lymphocytes	Black	189	1.0–3.0	428	1.0–3.4
	Nonblack	1,831	0.9–3.0	1980	1.0–3.4
Monocytes	Black	189	0.1–0.7	424	0.2–0.8
	Nonblack	1,831	0.2–0.9	1976	0.2–0.9
Eosinophils	Black	189	0.0–0.7	425	0.0–0.6
	Nonblack	1,832	0.0–0.6	1980	0.0–0.5
Basophils	Black	189	0.0–0.1	424	0.0–0.1
	Nonblack	1,831	0.0–0.1	1975	0.0–0.1

**Table 6:** Selection of reference intervals (RIs) from The Doctors Laboratory (TDL), hospitals, private clinics, and haematological charities within the UK.

Parameter 10^9^/liter	TDL	Royal Wolverhampton NHS trust	CLL support association	Cancer research UK	NHS Shetland	Gloucestershire Hospitals NHS trust	County Durham and Darlington NHS trust	Southwest London Pathology NHS	North Devon NHS trust	Maidstone and Tunbridge Wells NHS trust	Oxford University hospitals NHS trust	The London Clinic	Biobank	Royal Cornwall Hospitals NHS trust	Wakeman et al, 2007
WBC	3.0–10.0	4.0–11.0	3.8–10.8	4.0–11.0	4.0–11.0	3.6–11.0	4.0–11.0	4.0–11.0	4.0–11.0	3.4–11.0	4.0–11.0	4.0–11.0	3.53–9.57	3.7–11.1*	3.6–9.2
NEUT	2.0–7.5	2.0–7.5	2.0–7.5	2.0–7.5	2.0–7.0	1.8–7.5	1.7–7.5	1.7–8.0	1.8–7.5	1.7–8.0	2.0–7.0	2.0–7.5	1.47–7.06	1.7–7.5	1.7–6.2
LYMPH	1.2–3.7	1.5–4.5	1.3–3.5	1.0–4.5	1.0–3.5	1.0–4.0	1.5–4.5	1.0–4.0	1.0–3.5	1.0–4.0	1.0–3.0	1.5–4.0	0.65–4.25	1.0–3.2	1.0–3.4
MONO	0.2–1.0	0.2–0.8	n/a	n/a	0.2–1.0	0.2–0.8	0.2–1.0	0.24–1.1	0.1–1.0	0.2–1.5	0.2–1.0	0.4–1.0	0.17–1.21	0.2–0.6	0.2–0.8
EOSIN	0.0–0.4	0.0–0.4	n/a	n/a	0.0–0.5	0.1–0.4	0.0–0.5	0.1–0.8	0.04–0.4	0.0–0.5	0.02–0.5	0.04–0.4	0.03–0.77	0.1–0.5	0.0–0.4
BASO	0.0–0.1	0.0–0.1	n/a	n/a	0.0–0.3	0.02–0.1	0.0–0.1	0.0–0.3	0.0–0.25	0.0–0.1	0.02–0.1	0.0–0.1	0.01–0.13	0.02–0.1	0.0–0.1

*Male and female ranges combined.

n/a, not available; WBC, white blood cell; NEUT, neutrophils; LYMPH, lymphocytes; MONO-monocytes; EOSIN, eosinophils; BASO, basophils; TDL, The Doctors Laboratory.

For total WBC and neutrophils, the proposed lower limit of normal (LLN) and upper limit of normal (ULN) of the morning and evening ranges are lower for black volunteers, compared with nonblack volunteers. There is little variation between both populations with respect to lymphocyte ranges for the morning and evening periods. The proposed morning monocyte normal range for the black population is slightly lower than that of the nonblack population, and evening ranges are roughly the same for both populations. In the case of eosinophils, the proposed morning ULN is higher for black volunteers compared with nonblack volunteers; little variation is seen between the evening eosinophil ranges of both populations. Basophils morning and evening ranges are the same for both populations.

The ULN of the evening ranges was higher for total WBC count, neutrophils and lymphocytes when compared to the morning ranges for both populations, with the exception of eosinophils which had a lower ULN in the evening for black populations. The evening eosinophil range for nonblack populations showed an increase in the LLN relative to the morning range. No diurnal variation was seen in the ULN ranges for both population groups with respect to monocytes and basophils.

## Discussion

This study examined the effects of racial and diurnal variation upon total WBC counts and counts of individual WBC parameters (neutrophils, lymphocytes, eosinophils, basophils, and monocytes) in 7,157 ethnically diverse individuals from whom blood samples were obtained during the waking period (8 am to 6 pm). This study also proposed creating separate race and time-specific RIs for each WBC parameter. As no difference in the number of leukocytes was observed between Caucasian and Asian subjects, data from these two racial groups were pooled and these volunteers are referred to as nonblack subjects.

The existing literature indicates that individuals of African ancestry have lower WBC and neutrophil counts relative to individuals from other ethnic backgrounds (Chen et al., 2010) correlating with the results presented herein which demonstrated that volunteers from African backgrounds had lower total WBC, neutrophil, monocyte, basophil, and eosinophil counts relative to their Caucasian and Asian counterparts, but higher lymphocyte counts. Previous research has shown that both total WBC and its constituent cells (neutrophils, monocytes, lymphocytes, eosinophils) fluctuate in quantity throughout the day (Sennels et al., 2011; Pick et al., 2019). This is in line with this study’s findings which showed that the serum levels of all cell types were higher in the morning relative to the evening for both black and nonblack subjects, with the exception of eosinophils which showed the opposite trend in both groups. That is, eosinophils were higher in the morning than in the evening. This finding was statistically significant only for nonblack volunteers. One possible reason why differences in basophil diurnal variation were not significant for either black or nonblack subjects could be because these cells make up a tiny proportion of the total WBC count and are thus too few for any statistically significant effects to be observed.

### Biological Explanations and Consequences for the Observed Racial Differences

Cytokine-stimulated levels of circulating neutrophils are as numerous in people of indigenous African and Middle Eastern races as in individuals with European ancestry, although resting circulating neutrophil levels are lower in the former. The lower level is not pathogenic and is termed BEN.

The allele frequency giving rise to neutropenia in some groups of African and Middle Eastern origin has been attributed to a selection advantage due to an associated resistance to the development of malaria following *Plasmodium vivax* infection. Individuals diagnosed with BEN have a reduced number of bone marrow myeloid progenitors (Rezvani et al., 2001; Hollowell et al., 2005) and lower mature neutrophil levels (Mason et al., 1979). The change in WBC count over the day would suggest that the likelihood of a black subject failing to be included because of BEN is higher in the morning than later in the day since diurnal variation would further lower their WBC count. The mechanism of diurnal variation in neutrophil numbers is probably not related to the endogenous molecular clock (Ella et al., 2016; Wang et al., 2016). Neutrophils have a high turnover rate and cortisol inhibits their apoptosis (Zen et al., 2011). The diurnal variation seen in neutrophil count is the inverse of that expected if glucocorticoid secretion were responsible. Neutrophil levels are lowest when endogenous cortisol is at its daily maximum–cortisol levels reach a peak at around 08.30 am (Casanova-Acebes et al., 2013).

Another condition where individuals present with low leukocyte and neutrophil counts is termed benign familial leukopenia-neutropenia (BFLN) and has been reported in several racial groups, including Yemenite Jews, Blacks of South African extraction, West Indians and Arab Jordanians (Shoenfeld et al., 1988). Individuals with BFLN were shown not to have an increased incidence of infection, and their response to infection did not differ from subjects who had normal WBC (Shoenfeld et al., 1988).

Overall, mean lymphocyte counts were higher in black subjects than in nonblack subjects. Higher lymphocyte counts in black individuals can, in some circumstances, be indicative of infection or an autoimmune disorder. An increase in the number of lymphocytes is also associated with certain forms of lymphoma and leukaemia. These findings are consistent with previously published reports (Zelzulka et al., 1987; Cheng et al., 2004; Kaaba et al., 2004). To the contrary, some authors have found no difference in lymphocyte count variation between racial groups (Bain et al., 1984). A higher lymphocyte count in black subjects may be compensating for a lower neutrophil count and this phenomenon should be addressed in future research where prospective samples are analyzed.

### Potential Advantages of Using Race and Time-Specific RIs

The variations seen in the present study together with the recognised physiological differences between racial subpopulations and acknowledged racial differences in RIs for various haematological tests (Ash et al., 1983; Bain, 1996) show that for a multiracial country with a diverse population such as the United Kingdom, it is important to establish race-specific RIs. RIs developed using a mainly Caucasian population (Aytekin and Emerk, 2008; Lim et al., 2015) that fail to consider racial and diurnal variation, arguably lack validity and could result in clinicians being misled about any potential drug effects as well as the status of an individual’s immune system, resulting in their inappropriate exclusion from a clinical trial. It is thus more appropriate to establish ranges that take into account an individual’s racial background and the time of day. It is important to highlight that, in this study, the confidence intervals of the mean WBC counts did not overlap at any point throughout the day, indicating that black and nonblack populations have completely separate WBC distributions.

In comparison with The Doctors Laboratory (TDL) reference range that covers a wider population, the proposed intervals are generally narrower and the upper and lower limits of normal have, for most parameters, shifted. They, now, more accurately represent the normal ranges observed in a healthy racially diverse population.

The proposed RIs herein may impact clinical trials in several ways. Firstly, they may facilitate better representation of black racial groups in healthy volunteer trials. In the suggested RIs, the total WBC and neutrophil LLN for black subjects is lower than that of TDL, which may ensure that black subjects with a low neutrophil count normal for their racial group are not unnecessarily excluded from trials. Secondly, it may ensure that the recruitment of subjects into healthy volunteer trials is more representative of the local population. The ULN for total WBC and neutrophil counts in black subjects is also lower than in TDLs reference range. Therefore, a black subject with a morning WBC count of 10.0 × 10^9^/liter may currently be enrolled in a trial as this value is within the TDL reference range despite the value being 33% higher than the morning ULN normal for their race. Thus, the proposed RIs may allow better identification of subjects that should not be included in a healthy volunteer trial, at least until further clinical assessments have been conducted. Thirdly, more specific RIs may aid the development of rules governing postdose changes in WBC parameters. For example, an IMP that is expected to affect haematological parameters may require specific trial stopping rules concerning WBC counts. The race and time-specific RIs are generally narrower than TDL’s RIs, which could facilitate more accurate intra-subject monitoring of postdose changes. This offers several advantages for participant safety (e.g., quicker identification of drug-induced abnormalities and more accurate rules on when dosing cessation should be triggered) and ensures that trials are not stopped prematurely because values fall outside RIs that do not accommodate race or time of day. Fourthly, time-specific RIs may allow for greater attention being paid to the timing of blood samples being taken during clinical trials and enhanced comparability of results. Screening or admission blood tests may be taken at any time during the day, whereas postdose samples, like most clinical procedures after dosing, are often scheduled at a specific time and taken early in the morning. Often the untimed, predose samples are used as a baseline. Ensuring baseline and on-study haematology samples are collected at matching time points will aid better data interpretation and reporting.

Overall, the main benefit of race- and time-specific RIs is to facilitate accurate testing of drugs in development across a diverse population of healthy subjects and enable more precise postdose safety monitoring and reporting of clinical trials.

### Limitations of This Research

Limitations of this work included unbalanced subject demographics weighing towards white and Asian males, and sample collection between 8 am–6 pm that limited the analysis period to waking hours. It is likely that, in this analysis, the peak number of WBC counts was missed because diurnal peaks of various populations of WBCs may occur after 6 pm, as previously documented: eosinophils and basophils peak around midnight (12 am to 4 am) (Uhrbrand, 1958; Boseila, 1959; Dahl, 1977); monocyte counts peak at 8 pm (Haus et al., 1983), and neutrophils between 4 pm to 8 pm (Haus et al., 1983). However, trial participants are typically not screened for inclusion in study trials during these time periods. Nevertheless, this study was high-powered: it analyzed 13,332 blood samples from 7,157 healthy volunteers, adding significant weight to the body of evidence which indicates that WBC count parameters within healthy trial volunteers vary by time of day and race

Furthermore, blood samples were grouped according to the volunteers’ self-ascribed racial groups. As ascription of race is acknowledged as a poor marker for genetic diversity (Takezawa et al., 2014; Duster, 2015), it would be preferable but possibly impractical to group subjects according to the Duffy antigen allele they possess at the time of recruitment.

Lastly, the authors of this report are aware that the RIs proposed herein do not account for the differences in number of study subjects by ethnic group.

### Other Factors and Scope for Future Work

In addition to racial and diurnal variation in WBC parameters, the literature indicates that age, gender, smoking status (Hsieh et al., 2007), poor sleep and BMI (Nishitani and Dakakibara, 2007) may also affect neutrophil and total WBC counts. These factors should be taken into account for any prospective future studies investigating diurnal and racial differences in WBC parameters.

## Data Availability Statement

The datasets generated for this study are available on request to the corresponding author.

## Ethics Statement

Ethical review and approval was not required for the study on human participants in accordance with the local legislation and institutional requirements. Written informed consent for participation was not required for this study in accordance with the national legislation and the institutional requirements.

## Author Contributions

JT and UL designed the study. JT, UL, and SC were involved in subject recruitment for the different trials. All authors analyzed and interpreted the data. SC, TP, SF, DD and DW drafted the manuscript. All authors critically revised and approved the manuscript.

## Conflict of Interest

SC, TP, SF, DD, UL, and JT are employees of Richmond Pharmacology Ltd. DW is an employee of the Liverpool School of Tropical Medicine.
