# Supplementary material for: Time- and Race-Specific Haematological Reference Intervals for Healthy Volunteer Trials: A Retrospective Analysis of Pooled Data From Multiple Phase I Trials
Source: Front Pharmacol. 2020 Mar 13;11:314. doi: 10.3389/fphar.2020.00314 (PMC7082321; doi:10.3389/fphar.2020.00314)
Supplement: Supplementary file 1 [file Table_1.docx]

Table 1 GEE Model analysis of haematological parameters: Between and within group comparison

____________________________________________________________________________________________________

Label parameter=BASO(x10^8/L)

The GENMOD Procedure

Model Information

Data Set WORK.ENDPOINTS

Distribution Normal

Link Function Identity

Dependent Variable outcome

Number of Observations Read 12714

Number of Observations Used 12707

Missing Values 7

Class Level Information

Class Levels Values

study 75 RICH PHARM RP DFP RPC09003 RPC09004 RPC09008

RPC09029 RPC09051 RPC09052 RPC09054 RPC09068

RPC09069 RPC09070 RPC09071 RPC09079 RPC10009

RPC10021 RPC10027 RPC10035 RPC10045 RPC10050

RPC11002 RPC11003 RPC11035 RPC11050 RPC11061

RPC11067 RPC11069 RPC11089 ...

pat 7068 132 321 364 786 940 1213 2474 2535 2946 3040 3635

4026 4051 5656 5775 6017 6176 6646 6774 7094 8142

8560 8721 8805 8810 8924 9047 9051 9155 9624 10040

10308 10466 10563 10619 11604 11824 11980 12076

12431 12437 12712 13045 13575 13593 13946 14198

14404 ...

treat 2 A B

time 11 0 1 2 3 4 5 6 7 8 9 10

sex 2 F M

Fasted 2 N Y

Parameter Information

Parameter Effect treat time sex Fasted

Prm1 Intercept

Prm2 treat A

Prm3 treat B

Prm4 time 0

Prm5 time 1

Prm6 time 2

Prm7 time 3

Prm8 time 4

Prm9 time 5

Prm10 time 6

Prm11 time 7

Prm12 time 8

Prm13 time 9

Prm14 time 10

Prm15 treat*time A 0

Prm16 treat*time A 1

Prm17 treat*time A 2

Prm18 treat*time A 3

Prm19 treat*time A 4

Prm20 treat*time A 5

Prm21 treat*time A 6

Prm22 treat*time A 7

Prm23 treat*time A 8

Prm24 treat*time A 9

Prm25 treat*time A 10

Prm26 treat*time B 0

Prm27 treat*time B 1

____________________________________________________________________________________________________

A = Blacks, B = Non-blacks

Table 1 GEE Model analysis of haematological parameters: Between and within group comparison

____________________________________________________________________________________________________

Label parameter=BASO(x10^8/L)

The GENMOD Procedure

Parameter Information

Parameter Effect treat time sex Fasted

Prm28 treat*time B 2

Prm29 treat*time B 3

Prm30 treat*time B 4

Prm31 treat*time B 5

Prm32 treat*time B 6

Prm33 treat*time B 7

Prm34 treat*time B 8

Prm35 treat*time B 9

Prm36 treat*time B 10

Prm37 age

Prm38 sex F

Prm39 sex M

Prm40 Fasted N

Prm41 Fasted Y

Algorithm converged.

GEE Model Information

Correlation Structure Exchangeable

Subject Effect pat (7071 levels)

Number of Clusters 7071

Clusters With Missing Values 7

Correlation Matrix Dimension 20

Maximum Cluster Size 20

Minimum Cluster Size 0

Algorithm converged.

Exchangeable Working

Correlation

Correlation 0.7272894695

GEE Fit Criteria

QIC 12714.9013

QICu 12732.0000

Analysis Of GEE Parameter Estimates

Empirical Standard Error Estimates

Standard 95% Confidence

Parameter Estimate Error Limits Z Pr > |Z|

Intercept 0.2759 0.0111 0.2540 0.2977 24.76 <.0001

treat A -0.0434 0.0174 -0.0775 -0.0092 -2.49 0.0127

treat B 0.0000 0.0000 0.0000 0.0000 . .

time 0 -0.0092 0.0108 -0.0304 0.0120 -0.85 0.3945

time 1 -0.0174 0.0103 -0.0375 0.0028 -1.69 0.0909

time 2 -0.0148 0.0100 -0.0344 0.0048 -1.48 0.1391

time 3 -0.0126 0.0101 -0.0324 0.0072 -1.25 0.2126

time 4 -0.0156 0.0101 -0.0354 0.0042 -1.55 0.1221

time 5 -0.0190 0.0101 -0.0388 0.0008 -1.88 0.0604

time 6 -0.0072 0.0103 -0.0275 0.0130 -0.70 0.4845

time 7 -0.0154 0.0099 -0.0347 0.0040 -1.55 0.1205

____________________________________________________________________________________________________

A = Blacks, B = Non-blacks

Table 1 GEE Model analysis of haematological parameters: Between and within group comparison

____________________________________________________________________________________________________

Label parameter=BASO(x10^8/L)

The GENMOD Procedure

Analysis Of GEE Parameter Estimates

Empirical Standard Error Estimates

Standard 95% Confidence

Parameter Estimate Error Limits Z Pr > |Z|

time 8 -0.0136 0.0099 -0.0330 0.0057 -1.38 0.1680

time 9 -0.0101 0.0105 -0.0308 0.0106 -0.96 0.3378

time 10 0.0000 0.0000 0.0000 0.0000 . .

treat*time A 0 -0.0259 0.0283 -0.0814 0.0297 -0.91 0.3615

treat*time A 1 -0.0064 0.0195 -0.0447 0.0319 -0.33 0.7423

treat*time A 2 -0.0145 0.0193 -0.0524 0.0233 -0.75 0.4509

treat*time A 3 -0.0149 0.0195 -0.0531 0.0233 -0.76 0.4450

treat*time A 4 -0.0233 0.0194 -0.0614 0.0148 -1.20 0.2315

treat*time A 5 0.0064 0.0192 -0.0313 0.0440 0.33 0.7403

treat*time A 6 -0.0230 0.0184 -0.0591 0.0132 -1.25 0.2125

treat*time A 7 -0.0226 0.0188 -0.0594 0.0141 -1.21 0.2272

treat*time A 8 -0.0079 0.0182 -0.0437 0.0278 -0.43 0.6637

treat*time A 9 -0.0074 0.0193 -0.0453 0.0304 -0.39 0.6999

treat*time A 10 0.0000 0.0000 0.0000 0.0000 . .

treat*time B 0 0.0000 0.0000 0.0000 0.0000 . .

treat*time B 1 0.0000 0.0000 0.0000 0.0000 . .

treat*time B 2 0.0000 0.0000 0.0000 0.0000 . .

treat*time B 3 0.0000 0.0000 0.0000 0.0000 . .

treat*time B 4 0.0000 0.0000 0.0000 0.0000 . .

treat*time B 5 0.0000 0.0000 0.0000 0.0000 . .

treat*time B 6 0.0000 0.0000 0.0000 0.0000 . .

treat*time B 7 0.0000 0.0000 0.0000 0.0000 . .

treat*time B 8 0.0000 0.0000 0.0000 0.0000 . .

treat*time B 9 0.0000 0.0000 0.0000 0.0000 . .

treat*time B 10 0.0000 0.0000 0.0000 0.0000 . .

age 0.0009 0.0002 0.0006 0.0013 4.95 <.0001

sex F -0.0034 0.0041 -0.0114 0.0045 -0.85 0.3981

sex M 0.0000 0.0000 0.0000 0.0000 . .

Fasted N 0.0156 0.0076 0.0006 0.0305 2.04 0.0410

Fasted Y 0.0000 0.0000 0.0000 0.0000 . .

Contrast Estimate Results

Mean Mean L'Beta Standard

Label Estimate Confidence Limits Estimate Error Alpha

A vs B at 8 -0.0692 -0.1161 -0.0224 -0.0692 0.0239 0.05

A vs B at 9 -0.0498 -0.0718 -0.0279 -0.0498 0.0112 0.05

A vs B at 10 -0.0579 -0.0792 -0.0366 -0.0579 0.0109 0.05

A vs B at 11 -0.0583 -0.0847 -0.0318 -0.0583 0.0135 0.05

A vs B at 12 -0.0666 -0.0867 -0.0466 -0.0666 0.0102 0.05

A vs B at 13 -0.0370 -0.0590 -0.0151 -0.0370 0.0112 0.05

A vs B at 14 -0.0664 -0.0837 -0.0491 -0.0664 0.0088 0.05

A vs B at 15 -0.0660 -0.0840 -0.0481 -0.0660 0.0092 0.05

Contrast Estimate Results

L'Beta Chi-

Label Confidence Limits Square Pr > ChiSq

A vs B at 8 -0.1161 -0.0224 8.39 0.0038

A vs B at 9 -0.0718 -0.0279 19.80 <.0001

A vs B at 10 -0.0792 -0.0366 28.46 <.0001

A vs B at 11 -0.0847 -0.0318 18.63 <.0001

A vs B at 12 -0.0867 -0.0466 42.32 <.0001

A vs B at 13 -0.0590 -0.0151 10.93 0.0009

A vs B at 14 -0.0837 -0.0491 56.42 <.0001

A vs B at 15 -0.0840 -0.0481 51.85 <.0001

____________________________________________________________________________________________________

A = Blacks, B = Non-blacks

Table 1 GEE Model analysis of haematological parameters: Between and within group comparison

____________________________________________________________________________________________________

Label parameter=BASO(x10^8/L)

The GENMOD Procedure

Contrast Estimate Results

Mean Mean L'Beta Standard

Label Estimate Confidence Limits Estimate Error Alpha

A vs B at 16 -0.0513 -0.0687 -0.0340 -0.0513 0.0089 0.05

A vs B at 17 -0.0508 -0.0740 -0.0276 -0.0508 0.0118 0.05

A vs B at 18 -0.0434 -0.0775 -0.0092 -0.0434 0.0174 0.05

A vs B at 99 -0.0561 -0.0681 -0.0440 -0.0561 0.0061 0.05

Morning vs evening in A -0.0162 -0.0375 0.0051 -0.0162 0.0108 0.05

Morning vs evening in B -0.0058 -0.0149 0.0032 -0.0058 0.0046 0.05

Morning vs evening in X -0.0110 -0.0225 0.0005 -0.0110 0.0059 0.05

Contrast Estimate Results

L'Beta Chi-

Label Confidence Limits Square Pr > ChiSq

A vs B at 16 -0.0687 -0.0340 33.58 <.0001

A vs B at 17 -0.0740 -0.0276 18.47 <.0001

A vs B at 18 -0.0775 -0.0092 6.20 0.0127

A vs B at 99 -0.0681 -0.0440 83.45 <.0001

Morning vs evening in A -0.0375 0.0051 2.23 0.1352

Morning vs evening in B -0.0149 0.0032 1.58 0.2083

Morning vs evening in X -0.0225 0.0005 3.51 0.0611

treat*time Least Squares Means

Standard

treat time Estimate Error z Value Pr > |z| Alpha Lower Upper

A 0 0.2323 0.02342 9.92 <.0001 0.05 0.1863 0.2782

A 1 0.2435 0.01101 22.12 <.0001 0.05 0.2219 0.2651

A 2 0.2380 0.01068 22.28 <.0001 0.05 0.2170 0.2589

A 3 0.2398 0.01326 18.09 <.0001 0.05 0.2138 0.2658

A 4 0.2285 0.01027 22.24 <.0001 0.05 0.2083 0.2486

A 5 0.2547 0.01102 23.11 <.0001 0.05 0.2331 0.2763

A 6 0.2371 0.008635 27.46 <.0001 0.05 0.2202 0.2540

A 7 0.2293 0.008984 25.52 <.0001 0.05 0.2117 0.2469

A 8 0.2458 0.008674 28.34 <.0001 0.05 0.2288 0.2628

A 9 0.2498 0.01104 22.63 <.0001 0.05 0.2281 0.2714

A 10 0.2673 0.01475 18.13 <.0001 0.05 0.2384 0.2962

B 0 0.3015 0.006976 43.22 <.0001 0.05 0.2878 0.3152

B 1 0.2933 0.005812 50.47 <.0001 0.05 0.2819 0.3047

B 2 0.2959 0.005334 55.48 <.0001 0.05 0.2855 0.3064

B 3 0.2981 0.005463 54.57 <.0001 0.05 0.2874 0.3088

B 4 0.2951 0.005120 57.64 <.0001 0.05 0.2851 0.3051

B 5 0.2917 0.005415 53.87 <.0001 0.05 0.2811 0.3023

B 6 0.3035 0.005538 54.80 <.0001 0.05 0.2926 0.3143

B 7 0.2954 0.005227 56.50 <.0001 0.05 0.2851 0.3056

B 8 0.2971 0.005559 53.45 <.0001 0.05 0.2862 0.3080

B 9 0.3006 0.006306 47.67 <.0001 0.05 0.2882 0.3130

B 10 0.3107 0.01068 29.10 <.0001 0.05 0.2898 0.3316

____________________________________________________________________________________________________

A = Blacks, B = Non-blacks

Table 1 GEE Model analysis of haematological parameters: Between and within group comparison

____________________________________________________________________________________________________

Label parameter=EOSI(x10^8/L)

The GENMOD Procedure

Model Information

Data Set WORK.ENDPOINTS

Distribution Normal

Link Function Identity

Dependent Variable outcome

Number of Observations Read 12734

Number of Observations Used 12727

Missing Values 7

Class Level Information

Class Levels Values

study 75 RICH PHARM RP DFP RPC09003 RPC09004 RPC09008

RPC09029 RPC09051 RPC09052 RPC09054 RPC09068

RPC09069 RPC09070 RPC09071 RPC09079 RPC10009

RPC10021 RPC10027 RPC10035 RPC10045 RPC10050

RPC11002 RPC11003 RPC11035 RPC11050 RPC11061

RPC11067 RPC11069 RPC11089 ...

pat 7068 132 321 364 786 940 1213 2474 2535 2946 3040 3635

4026 4051 5656 5775 6017 6176 6646 6774 7094 8142

8560 8721 8805 8810 8924 9047 9051 9155 9624 10040

10308 10466 10563 10619 11604 11824 11980 12076

12431 12437 12712 13045 13575 13593 13946 14198

14404 ...

treat 2 A B

time 11 0 1 2 3 4 5 6 7 8 9 10

sex 2 F M

Fasted 2 N Y

Parameter Information

Parameter Effect treat time sex Fasted

Prm1 Intercept

Prm2 treat A

Prm3 treat B

Prm4 time 0

Prm5 time 1

Prm6 time 2

Prm7 time 3

Prm8 time 4

Prm9 time 5

Prm10 time 6

Prm11 time 7

Prm12 time 8

Prm13 time 9

Prm14 time 10

Prm15 treat*time A 0

Prm16 treat*time A 1

Prm17 treat*time A 2

Prm18 treat*time A 3

Prm19 treat*time A 4

Prm20 treat*time A 5

Prm21 treat*time A 6

Prm22 treat*time A 7

Prm23 treat*time A 8

Prm24 treat*time A 9

Prm25 treat*time A 10

Prm26 treat*time B 0

Prm27 treat*time B 1

____________________________________________________________________________________________________

A = Blacks, B = Non-blacks

Table 1 GEE Model analysis of haematological parameters: Between and within group comparison

____________________________________________________________________________________________________

Label parameter=EOSI(x10^8/L)

The GENMOD Procedure

Parameter Information

Parameter Effect treat time sex Fasted

Prm28 treat*time B 2

Prm29 treat*time B 3

Prm30 treat*time B 4

Prm31 treat*time B 5

Prm32 treat*time B 6

Prm33 treat*time B 7

Prm34 treat*time B 8

Prm35 treat*time B 9

Prm36 treat*time B 10

Prm37 age

Prm38 sex F

Prm39 sex M

Prm40 Fasted N

Prm41 Fasted Y

Algorithm converged.

GEE Model Information

Correlation Structure Exchangeable

Subject Effect pat (7071 levels)

Number of Clusters 7071

Clusters With Missing Values 7

Correlation Matrix Dimension 20

Maximum Cluster Size 20

Minimum Cluster Size 0

Algorithm converged.

Exchangeable Working

Correlation

Correlation 0.9999

GEE Fit Criteria

QIC 12950.1389

QICu 12752.0000

Analysis Of GEE Parameter Estimates

Empirical Standard Error Estimates

Standard 95% Confidence

Parameter Estimate Error Limits Z Pr > |Z|

Intercept 0.8621 0.4414 -0.0030 1.7273 1.95 0.0508

treat A -0.2988 0.1258 -0.5454 -0.0522 -2.37 0.0176

treat B 0.0000 0.0000 0.0000 0.0000 . .

time 0 0.2426 0.0960 0.0545 0.4307 2.53 0.0115

time 1 0.0771 0.0883 -0.0959 0.2501 0.87 0.3824

time 2 -0.0210 0.0917 -0.2007 0.1587 -0.23 0.8186

time 3 -0.1877 0.0955 -0.3750 -0.0004 -1.96 0.0495

time 4 -0.2095 0.0902 -0.3864 -0.0327 -2.32 0.0202

time 5 -0.2121 0.0905 -0.3895 -0.0346 -2.34 0.0192

time 6 -0.2661 0.0947 -0.4517 -0.0806 -2.81 0.0049

time 7 -0.2672 0.0923 -0.4482 -0.0863 -2.89 0.0038

____________________________________________________________________________________________________

A = Blacks, B = Non-blacks

Table 1 GEE Model analysis of haematological parameters: Between and within group comparison

____________________________________________________________________________________________________

Label parameter=EOSI(x10^8/L)

The GENMOD Procedure

Analysis Of GEE Parameter Estimates

Empirical Standard Error Estimates

Standard 95% Confidence

Parameter Estimate Error Limits Z Pr > |Z|

time 8 -0.2632 0.0949 -0.4493 -0.0771 -2.77 0.0056

time 9 -0.1904 0.0986 -0.3836 0.0028 -1.93 0.0534

time 10 0.0000 0.0000 0.0000 0.0000 . .

treat*time A 0 -0.1372 0.3914 -0.9044 0.6301 -0.35 0.7260

treat*time A 1 0.1003 0.1575 -0.2084 0.4090 0.64 0.5242

treat*time A 2 0.1137 0.1496 -0.1795 0.4070 0.76 0.4472

treat*time A 3 0.4039 0.2149 -0.0174 0.8251 1.88 0.0602

treat*time A 4 0.0092 0.1525 -0.2896 0.3081 0.06 0.9517

treat*time A 5 0.1384 0.1483 -0.1522 0.4291 0.93 0.3505

treat*time A 6 0.2621 0.1520 -0.0358 0.5600 1.72 0.0847

treat*time A 7 0.1618 0.1422 -0.1169 0.4404 1.14 0.2552

treat*time A 8 0.1956 0.1406 -0.0800 0.4713 1.39 0.1642

treat*time A 9 0.3486 0.2008 -0.0450 0.7422 1.74 0.0826

treat*time A 10 0.0000 0.0000 0.0000 0.0000 . .

treat*time B 0 0.0000 0.0000 0.0000 0.0000 . .

treat*time B 1 0.0000 0.0000 0.0000 0.0000 . .

treat*time B 2 0.0000 0.0000 0.0000 0.0000 . .

treat*time B 3 0.0000 0.0000 0.0000 0.0000 . .

treat*time B 4 0.0000 0.0000 0.0000 0.0000 . .

treat*time B 5 0.0000 0.0000 0.0000 0.0000 . .

treat*time B 6 0.0000 0.0000 0.0000 0.0000 . .

treat*time B 7 0.0000 0.0000 0.0000 0.0000 . .

treat*time B 8 0.0000 0.0000 0.0000 0.0000 . .

treat*time B 9 0.0000 0.0000 0.0000 0.0000 . .

treat*time B 10 0.0000 0.0000 0.0000 0.0000 . .

age 0.0315 0.0143 0.0035 0.0594 2.21 0.0273

sex F -0.2638 0.0418 -0.3457 -0.1820 -6.32 <.0001

sex M 0.0000 0.0000 0.0000 0.0000 . .

Fasted N 0.3152 0.0867 0.1453 0.4852 3.64 0.0003

Fasted Y 0.0000 0.0000 0.0000 0.0000 . .

Contrast Estimate Results

Mean Mean L'Beta Standard

Label Estimate Confidence Limits Estimate Error Alpha

A vs B at 8 -0.4360 -1.1678 0.2958 -0.4360 0.3734 0.05

A vs B at 9 -0.1985 -0.4202 0.0232 -0.1985 0.1131 0.05

A vs B at 10 -0.1851 -0.3767 0.0066 -0.1851 0.0978 0.05

A vs B at 11 0.1051 -0.2831 0.4933 0.1051 0.1981 0.05

A vs B at 12 -0.2896 -0.5128 -0.0663 -0.2896 0.1139 0.05

A vs B at 13 -0.1604 -0.3722 0.0514 -0.1604 0.1081 0.05

A vs B at 14 -0.0367 -0.2351 0.1617 -0.0367 0.1012 0.05

A vs B at 15 -0.1370 -0.3060 0.0319 -0.1370 0.0862 0.05

Contrast Estimate Results

L'Beta Chi-

Label Confidence Limits Square Pr > ChiSq

A vs B at 8 -1.1678 0.2958 1.36 0.2430

A vs B at 9 -0.4202 0.0232 3.08 0.0793

A vs B at 10 -0.3767 0.0066 3.58 0.0584

A vs B at 11 -0.2831 0.4933 0.28 0.5957

A vs B at 12 -0.5128 -0.0663 6.46 0.0110

A vs B at 13 -0.3722 0.0514 2.20 0.1378

A vs B at 14 -0.2351 0.1617 0.13 0.7168

A vs B at 15 -0.3060 0.0319 2.53 0.1119

____________________________________________________________________________________________________

A = Blacks, B = Non-blacks

Table 1 GEE Model analysis of haematological parameters: Between and within group comparison

____________________________________________________________________________________________________

Label parameter=EOSI(x10^8/L)

The GENMOD Procedure

Contrast Estimate Results

Mean Mean L'Beta Standard

Label Estimate Confidence Limits Estimate Error Alpha

A vs B at 16 -0.1032 -0.2688 0.0624 -0.1032 0.0845 0.05

A vs B at 17 0.0498 -0.3024 0.4020 0.0498 0.1797 0.05

A vs B at 18 -0.2988 -0.5454 -0.0522 -0.2988 0.1258 0.05

A vs B at 99 -0.1537 -0.2701 -0.0372 -0.1537 0.0594 0.05

Morning vs evening in A 0.0940 -0.2873 0.4754 0.0940 0.1946 0.05

Morning vs evening in B 0.2483 0.1658 0.3307 0.2483 0.0421 0.05

Morning vs evening in X 0.1711 -0.0209 0.3631 0.1711 0.0980 0.05

Contrast Estimate Results

L'Beta Chi-

Label Confidence Limits Square Pr > ChiSq

A vs B at 16 -0.2688 0.0624 1.49 0.2220

A vs B at 17 -0.3024 0.4020 0.08 0.7815

A vs B at 18 -0.5454 -0.0522 5.64 0.0176

A vs B at 99 -0.2701 -0.0372 6.69 0.0097

Morning vs evening in A -0.2873 0.4754 0.23 0.6289

Morning vs evening in B 0.1658 0.3307 34.84 <.0001

Morning vs evening in X -0.0209 0.3631 3.05 0.0806

treat*time Least Squares Means

Standard

treat time Estimate Error z Value Pr > |z| Alpha Lower Upper

A 0 1.6865 0.3487 4.84 <.0001 0.05 1.0031 2.3699

A 1 1.7585 0.1045 16.83 <.0001 0.05 1.5537 1.9633

A 2 1.6738 0.08852 18.91 <.0001 0.05 1.5003 1.8473

A 3 1.7973 0.2174 8.27 <.0001 0.05 1.3711 2.2234

A 4 1.3808 0.09763 14.14 <.0001 0.05 1.1894 1.5721

A 5 1.5075 0.1147 13.15 <.0001 0.05 1.2828 1.7322

A 6 1.5771 0.1068 14.77 <.0001 0.05 1.3678 1.7863

A 7 1.4756 0.08498 17.36 <.0001 0.05 1.3090 1.6422

A 8 1.5135 0.07475 20.25 <.0001 0.05 1.3670 1.6600

A 9 1.7393 0.1934 8.99 <.0001 0.05 1.3603 2.1183

A 10 1.5811 0.09800 16.13 <.0001 0.05 1.3890 1.7732

B 0 2.1225 0.06655 31.89 <.0001 0.05 1.9921 2.2529

B 1 1.9570 0.05728 34.17 <.0001 0.05 1.8447 2.0693

B 2 1.8589 0.05565 33.40 <.0001 0.05 1.7498 1.9680

B 3 1.6922 0.06370 26.56 <.0001 0.05 1.5673 1.8170

B 4 1.6704 0.05428 30.77 <.0001 0.05 1.5640 1.7767

B 5 1.6678 0.05589 29.84 <.0001 0.05 1.5583 1.7774

B 6 1.6138 0.05916 27.28 <.0001 0.05 1.4978 1.7297

B 7 1.6127 0.05298 30.44 <.0001 0.05 1.5088 1.7165

B 8 1.6167 0.05609 28.82 <.0001 0.05 1.5067 1.7266

B 9 1.6895 0.05910 28.58 <.0001 0.05 1.5736 1.8053

B 10 1.8799 0.09557 19.67 <.0001 0.05 1.6926 2.0672

____________________________________________________________________________________________________

A = Blacks, B = Non-blacks

Table 1 GEE Model analysis of haematological parameters: Between and within group comparison

____________________________________________________________________________________________________

Label parameter=LYMP(x10^8/L)

The GENMOD Procedure

Model Information

Data Set WORK.ENDPOINTS

Distribution Normal

Link Function Identity

Dependent Variable outcome

Number of Observations Read 12744

Number of Observations Used 12737

Missing Values 7

Class Level Information

Class Levels Values

study 75 RICH PHARM RP DFP RPC09003 RPC09004 RPC09008

RPC09029 RPC09051 RPC09052 RPC09054 RPC09068

RPC09069 RPC09070 RPC09071 RPC09079 RPC10009

RPC10021 RPC10027 RPC10035 RPC10045 RPC10050

RPC11002 RPC11003 RPC11035 RPC11050 RPC11061

RPC11067 RPC11069 RPC11089 ...

pat 7068 132 321 364 786 940 1213 2474 2535 2946 3040 3635

4026 4051 5656 5775 6017 6176 6646 6774 7094 8142

8560 8721 8805 8810 8924 9047 9051 9155 9624 10040

10308 10466 10563 10619 11604 11824 11980 12076

12431 12437 12712 13045 13575 13593 13946 14198

14404 ...

treat 2 A B

time 11 0 1 2 3 4 5 6 7 8 9 10

sex 2 F M

Fasted 2 N Y

Parameter Information

Parameter Effect treat time sex Fasted

Prm1 Intercept

Prm2 treat A

Prm3 treat B

Prm4 time 0

Prm5 time 1

Prm6 time 2

Prm7 time 3

Prm8 time 4

Prm9 time 5

Prm10 time 6

Prm11 time 7

Prm12 time 8

Prm13 time 9

Prm14 time 10

Prm15 treat*time A 0

Prm16 treat*time A 1

Prm17 treat*time A 2

Prm18 treat*time A 3

Prm19 treat*time A 4

Prm20 treat*time A 5

Prm21 treat*time A 6

Prm22 treat*time A 7

Prm23 treat*time A 8

Prm24 treat*time A 9

Prm25 treat*time A 10

Prm26 treat*time B 0

Prm27 treat*time B 1

____________________________________________________________________________________________________

A = Blacks, B = Non-blacks

Table 1 GEE Model analysis of haematological parameters: Between and within group comparison

____________________________________________________________________________________________________

Label parameter=LYMP(x10^8/L)

The GENMOD Procedure

Parameter Information

Parameter Effect treat time sex Fasted

Prm28 treat*time B 2

Prm29 treat*time B 3

Prm30 treat*time B 4

Prm31 treat*time B 5

Prm32 treat*time B 6

Prm33 treat*time B 7

Prm34 treat*time B 8

Prm35 treat*time B 9

Prm36 treat*time B 10

Prm37 age

Prm38 sex F

Prm39 sex M

Prm40 Fasted N

Prm41 Fasted Y

Algorithm converged.

GEE Model Information

Correlation Structure Exchangeable

Subject Effect pat (7071 levels)

Number of Clusters 7071

Clusters With Missing Values 7

Correlation Matrix Dimension 20

Maximum Cluster Size 20

Minimum Cluster Size 0

Algorithm converged.

Exchangeable Working

Correlation

Correlation 0.6080944871

GEE Fit Criteria

QIC 12750.5306

QICu 12762.0000

Analysis Of GEE Parameter Estimates

Empirical Standard Error Estimates

Standard 95% Confidence

Parameter Estimate Error Limits Z Pr > |Z|

Intercept 21.5376 0.3850 20.7829 22.2922 55.94 <.0001

treat A 0.7999 0.6271 -0.4292 2.0291 1.28 0.2021

treat B 0.0000 0.0000 0.0000 0.0000 . .

time 0 -2.6134 0.3957 -3.3890 -1.8378 -6.60 <.0001

time 1 -3.4931 0.3523 -4.1835 -2.8027 -9.92 <.0001

time 2 -3.6731 0.3458 -4.3509 -2.9953 -10.62 <.0001

time 3 -4.2049 0.3404 -4.8721 -3.5377 -12.35 <.0001

time 4 -3.5689 0.3417 -4.2386 -2.8992 -10.45 <.0001

time 5 -3.3125 0.3419 -3.9827 -2.6423 -9.69 <.0001

time 6 -2.3864 0.3499 -3.0723 -1.7006 -6.82 <.0001

time 7 -1.8565 0.3462 -2.5350 -1.1780 -5.36 <.0001

____________________________________________________________________________________________________

A = Blacks, B = Non-blacks

Table 1 GEE Model analysis of haematological parameters: Between and within group comparison

____________________________________________________________________________________________________

Label parameter=LYMP(x10^8/L)

The GENMOD Procedure

Analysis Of GEE Parameter Estimates

Empirical Standard Error Estimates

Standard 95% Confidence

Parameter Estimate Error Limits Z Pr > |Z|

time 8 -1.3428 0.3463 -2.0215 -0.6640 -3.88 0.0001

time 9 -0.8936 0.3667 -1.6124 -0.1748 -2.44 0.0148

time 10 0.0000 0.0000 0.0000 0.0000 . .

treat*time A 0 0.7565 0.9725 -1.1496 2.6625 0.78 0.4366

treat*time A 1 0.0455 0.7511 -1.4266 1.5176 0.06 0.9517

treat*time A 2 -0.7649 0.7009 -2.1387 0.6088 -1.09 0.2751

treat*time A 3 0.3758 0.7072 -1.0102 1.7618 0.53 0.5951

treat*time A 4 -0.3229 0.6660 -1.6283 0.9825 -0.48 0.6278

treat*time A 5 -0.7486 0.6989 -2.1184 0.6213 -1.07 0.2842

treat*time A 6 -0.8078 0.6841 -2.1487 0.5331 -1.18 0.2377

treat*time A 7 -0.5139 0.6752 -1.8373 0.8096 -0.76 0.4467

treat*time A 8 -0.7388 0.6713 -2.0545 0.5770 -1.10 0.2711

treat*time A 9 -0.2046 0.7153 -1.6066 1.1973 -0.29 0.7748

treat*time A 10 0.0000 0.0000 0.0000 0.0000 . .

treat*time B 0 0.0000 0.0000 0.0000 0.0000 . .

treat*time B 1 0.0000 0.0000 0.0000 0.0000 . .

treat*time B 2 0.0000 0.0000 0.0000 0.0000 . .

treat*time B 3 0.0000 0.0000 0.0000 0.0000 . .

treat*time B 4 0.0000 0.0000 0.0000 0.0000 . .

treat*time B 5 0.0000 0.0000 0.0000 0.0000 . .

treat*time B 6 0.0000 0.0000 0.0000 0.0000 . .

treat*time B 7 0.0000 0.0000 0.0000 0.0000 . .

treat*time B 8 0.0000 0.0000 0.0000 0.0000 . .

treat*time B 9 0.0000 0.0000 0.0000 0.0000 . .

treat*time B 10 0.0000 0.0000 0.0000 0.0000 . .

age 0.0246 0.0062 0.0124 0.0368 3.95 <.0001

sex F 1.2837 0.1347 1.0198 1.5476 9.53 <.0001

sex M 0.0000 0.0000 0.0000 0.0000 . .

Fasted N 0.3699 0.2286 -0.0782 0.8180 1.62 0.1057

Fasted Y 0.0000 0.0000 0.0000 0.0000 . .

Contrast Estimate Results

Mean Mean L'Beta Standard

Label Estimate Confidence Limits Estimate Error Alpha

A vs B at 8 1.5564 0.0959 3.0169 1.5564 0.7452 0.05

A vs B at 9 0.8455 -0.0456 1.7366 0.8455 0.4546 0.05

A vs B at 10 0.0350 -0.6542 0.7243 0.0350 0.3517 0.05

A vs B at 11 1.1758 0.3753 1.9762 1.1758 0.4084 0.05

A vs B at 12 0.4770 -0.1991 1.1532 0.4770 0.3450 0.05

A vs B at 13 0.0514 -0.5840 0.6868 0.0514 0.3242 0.05

A vs B at 14 -0.0079 -0.6517 0.6360 -0.0079 0.3285 0.05

A vs B at 15 0.2861 -0.4096 0.9818 0.2861 0.3550 0.05

Contrast Estimate Results

L'Beta Chi-

Label Confidence Limits Square Pr > ChiSq

A vs B at 8 0.0959 3.0169 4.36 0.0367

A vs B at 9 -0.0456 1.7366 3.46 0.0629

A vs B at 10 -0.6542 0.7243 0.01 0.9207

A vs B at 11 0.3753 1.9762 8.29 0.0040

A vs B at 12 -0.1991 1.1532 1.91 0.1667

A vs B at 13 -0.5840 0.6868 0.03 0.8741

A vs B at 14 -0.6517 0.6360 0.00 0.9809

A vs B at 15 -0.4096 0.9818 0.65 0.4203

____________________________________________________________________________________________________

A = Blacks, B = Non-blacks

Table 1 GEE Model analysis of haematological parameters: Between and within group comparison

____________________________________________________________________________________________________

Label parameter=LYMP(x10^8/L)

The GENMOD Procedure

Contrast Estimate Results

Mean Mean L'Beta Standard

Label Estimate Confidence Limits Estimate Error Alpha

A vs B at 16 0.0612 -0.6069 0.7293 0.0612 0.3409 0.05

A vs B at 17 0.5953 -0.2466 1.4373 0.5953 0.4296 0.05

A vs B at 18 0.7999 -0.4292 2.0291 0.7999 0.6271 0.05

A vs B at 99 0.5342 0.1382 0.9301 0.5342 0.2020 0.05

Morning vs evening in A -2.1657 -2.9232 -1.4082 -2.1657 0.3865 0.05

Morning vs evening in B -2.4893 -2.8167 -2.1618 -2.4893 0.1671 0.05

Morning vs evening in X -2.3275 -2.7402 -1.9148 -2.3275 0.2106 0.05

Contrast Estimate Results

L'Beta Chi-

Label Confidence Limits Square Pr > ChiSq

A vs B at 16 -0.6069 0.7293 0.03 0.8576

A vs B at 17 -0.2466 1.4373 1.92 0.1658

A vs B at 18 -0.4292 2.0291 1.63 0.2021

A vs B at 99 0.1382 0.9301 6.99 0.0082

Morning vs evening in A -2.9232 -1.4082 31.40 <.0001

Morning vs evening in B -2.8167 -2.1618 222.03 <.0001

Morning vs evening in X -2.7402 -1.9148 122.17 <.0001

treat*time Least Squares Means

Standard

treat time Estimate Error z Value Pr > |z| Alpha Lower Upper

A 0 22.0830 0.7138 30.94 <.0001 0.05 20.6841 23.4820

A 1 20.4924 0.4458 45.96 <.0001 0.05 19.6186 21.3662

A 2 19.5019 0.3492 55.84 <.0001 0.05 18.8174 20.1864

A 3 20.1109 0.4068 49.43 <.0001 0.05 19.3135 20.9082

A 4 20.0481 0.3419 58.63 <.0001 0.05 19.3780 20.7183

A 5 19.8789 0.3236 61.42 <.0001 0.05 19.2446 20.5132

A 6 20.7457 0.3131 66.26 <.0001 0.05 20.1321 21.3594

A 7 21.5696 0.3432 62.85 <.0001 0.05 20.8969 22.2423

A 8 21.8584 0.3318 65.89 <.0001 0.05 21.2082 22.5086

A 9 22.8417 0.3953 57.78 <.0001 0.05 22.0669 23.6165

A 10 23.9399 0.5424 44.14 <.0001 0.05 22.8770 25.0029

B 0 20.5266 0.2540 80.81 <.0001 0.05 20.0288 21.0245

B 1 19.6469 0.1836 106.99 <.0001 0.05 19.2870 20.0068

B 2 19.4669 0.1672 116.44 <.0001 0.05 19.1392 19.7946

B 3 18.9351 0.1692 111.89 <.0001 0.05 18.6034 19.2668

B 4 19.5711 0.1634 119.76 <.0001 0.05 19.2508 19.8914

B 5 19.8275 0.1603 123.67 <.0001 0.05 19.5133 20.1417

B 6 20.7536 0.1753 118.40 <.0001 0.05 20.4100 21.0971

B 7 21.2835 0.1790 118.87 <.0001 0.05 20.9326 21.6344

B 8 21.7972 0.1805 120.76 <.0001 0.05 21.4435 22.1510

B 9 22.2464 0.2132 104.32 <.0001 0.05 21.8284 22.6644

B 10 23.1400 0.3433 67.41 <.0001 0.05 22.4672 23.8128

____________________________________________________________________________________________________

A = Blacks, B = Non-blacks

Table 1 GEE Model analysis of haematological parameters: Between and within group comparison

____________________________________________________________________________________________________

Label parameter=MONO(x10^8/L)

The GENMOD Procedure

Model Information

Data Set WORK.ENDPOINTS

Distribution Normal

Link Function Identity

Dependent Variable outcome

Number of Observations Read 12718

Number of Observations Used 12711

Missing Values 7

Class Level Information

Class Levels Values

study 75 RICH PHARM RP DFP RPC09003 RPC09004 RPC09008

RPC09029 RPC09051 RPC09052 RPC09054 RPC09068

RPC09069 RPC09070 RPC09071 RPC09079 RPC10009

RPC10021 RPC10027 RPC10035 RPC10045 RPC10050

RPC11002 RPC11003 RPC11035 RPC11050 RPC11061

RPC11067 RPC11069 RPC11089 ...

pat 7068 132 321 364 786 940 1213 2474 2535 2946 3040 3635

4026 4051 5656 5775 6017 6176 6646 6774 7094 8142

8560 8721 8805 8810 8924 9047 9051 9155 9624 10040

10308 10466 10563 10619 11604 11824 11980 12076

12431 12437 12712 13045 13575 13593 13946 14198

14404 ...

treat 2 A B

time 11 0 1 2 3 4 5 6 7 8 9 10

sex 2 F M

Fasted 2 N Y

Parameter Information

Parameter Effect treat time sex Fasted

Prm1 Intercept

Prm2 treat A

Prm3 treat B

Prm4 time 0

Prm5 time 1

Prm6 time 2

Prm7 time 3

Prm8 time 4

Prm9 time 5

Prm10 time 6

Prm11 time 7

Prm12 time 8

Prm13 time 9

Prm14 time 10

Prm15 treat*time A 0

Prm16 treat*time A 1

Prm17 treat*time A 2

Prm18 treat*time A 3

Prm19 treat*time A 4

Prm20 treat*time A 5

Prm21 treat*time A 6

Prm22 treat*time A 7

Prm23 treat*time A 8

Prm24 treat*time A 9

Prm25 treat*time A 10

Prm26 treat*time B 0

Prm27 treat*time B 1

____________________________________________________________________________________________________

A = Blacks, B = Non-blacks

Table 1 GEE Model analysis of haematological parameters: Between and within group comparison

____________________________________________________________________________________________________

Label parameter=MONO(x10^8/L)

The GENMOD Procedure

Parameter Information

Parameter Effect treat time sex Fasted

Prm28 treat*time B 2

Prm29 treat*time B 3

Prm30 treat*time B 4

Prm31 treat*time B 5

Prm32 treat*time B 6

Prm33 treat*time B 7

Prm34 treat*time B 8

Prm35 treat*time B 9

Prm36 treat*time B 10

Prm37 age

Prm38 sex F

Prm39 sex M

Prm40 Fasted N

Prm41 Fasted Y

Algorithm converged.

GEE Model Information

Correlation Structure Exchangeable

Subject Effect pat (7071 levels)

Number of Clusters 7071

Clusters With Missing Values 7

Correlation Matrix Dimension 21

Maximum Cluster Size 21

Minimum Cluster Size 0

Algorithm converged.

Exchangeable Working

Correlation

Correlation 0.6654180142

GEE Fit Criteria

QIC 12724.1342

QICu 12736.0000

Analysis Of GEE Parameter Estimates

Empirical Standard Error Estimates

Standard 95% Confidence

Parameter Estimate Error Limits Z Pr > |Z|

Intercept 5.7510 0.1038 5.5476 5.9544 55.42 <.0001

treat A -0.9745 0.1677 -1.3033 -0.6458 -5.81 <.0001

treat B 0.0000 0.0000 0.0000 0.0000 . .

time 0 -0.5027 0.1100 -0.7183 -0.2871 -4.57 <.0001

time 1 -0.6425 0.1019 -0.8423 -0.4427 -6.30 <.0001

time 2 -0.6803 0.0974 -0.8713 -0.4894 -6.98 <.0001

time 3 -0.8499 0.1007 -1.0472 -0.6527 -8.44 <.0001

time 4 -0.8060 0.0978 -0.9976 -0.6144 -8.24 <.0001

time 5 -0.7835 0.0964 -0.9724 -0.5947 -8.13 <.0001

time 6 -0.6175 0.0970 -0.8077 -0.4273 -6.36 <.0001

time 7 -0.4099 0.0954 -0.5969 -0.2228 -4.29 <.0001

____________________________________________________________________________________________________

A = Blacks, B = Non-blacks

Table 1 GEE Model analysis of haematological parameters: Between and within group comparison

____________________________________________________________________________________________________

Label parameter=MONO(x10^8/L)

The GENMOD Procedure

Analysis Of GEE Parameter Estimates

Empirical Standard Error Estimates

Standard 95% Confidence

Parameter Estimate Error Limits Z Pr > |Z|

time 8 -0.3563 0.0951 -0.5427 -0.1698 -3.75 0.0002

time 9 -0.2873 0.1043 -0.4918 -0.0828 -2.75 0.0059

time 10 0.0000 0.0000 0.0000 0.0000 . .

treat*time A 0 0.1287 0.2616 -0.3840 0.6414 0.49 0.6227

treat*time A 1 0.2701 0.2114 -0.1441 0.6844 1.28 0.2012

treat*time A 2 0.0674 0.1827 -0.2907 0.4256 0.37 0.7121

treat*time A 3 0.2456 0.1927 -0.1321 0.6233 1.27 0.2025

treat*time A 4 0.2324 0.1903 -0.1406 0.6054 1.22 0.2220

treat*time A 5 0.1091 0.1830 -0.2496 0.4679 0.60 0.5511

treat*time A 6 0.0529 0.1837 -0.3071 0.4129 0.29 0.7734

treat*time A 7 -0.0583 0.1888 -0.4283 0.3118 -0.31 0.7576

treat*time A 8 -0.0049 0.1763 -0.3505 0.3407 -0.03 0.9777

treat*time A 9 0.1846 0.2026 -0.2126 0.5818 0.91 0.3624

treat*time A 10 0.0000 0.0000 0.0000 0.0000 . .

treat*time B 0 0.0000 0.0000 0.0000 0.0000 . .

treat*time B 1 0.0000 0.0000 0.0000 0.0000 . .

treat*time B 2 0.0000 0.0000 0.0000 0.0000 . .

treat*time B 3 0.0000 0.0000 0.0000 0.0000 . .

treat*time B 4 0.0000 0.0000 0.0000 0.0000 . .

treat*time B 5 0.0000 0.0000 0.0000 0.0000 . .

treat*time B 6 0.0000 0.0000 0.0000 0.0000 . .

treat*time B 7 0.0000 0.0000 0.0000 0.0000 . .

treat*time B 8 0.0000 0.0000 0.0000 0.0000 . .

treat*time B 9 0.0000 0.0000 0.0000 0.0000 . .

treat*time B 10 0.0000 0.0000 0.0000 0.0000 . .

age 0.0059 0.0017 0.0025 0.0093 3.40 0.0007

sex F -0.3604 0.0375 -0.4339 -0.2868 -9.61 <.0001

sex M 0.0000 0.0000 0.0000 0.0000 . .

Fasted N 0.3247 0.0819 0.1642 0.4853 3.96 <.0001

Fasted Y 0.0000 0.0000 0.0000 0.0000 . .

Contrast Estimate Results

Mean Mean L'Beta Standard

Label Estimate Confidence Limits Estimate Error Alpha

A vs B at 8 -0.8459 -1.2272 -0.4645 -0.8459 0.1946 0.05

A vs B at 9 -0.7044 -1.0003 -0.4085 -0.7044 0.1510 0.05

A vs B at 10 -0.9071 -1.1128 -0.7014 -0.9071 0.1049 0.05

A vs B at 11 -0.7289 -0.9507 -0.5072 -0.7289 0.1131 0.05

A vs B at 12 -0.7422 -0.9466 -0.5377 -0.7422 0.1043 0.05

A vs B at 13 -0.8654 -1.0607 -0.6702 -0.8654 0.0996 0.05

A vs B at 14 -0.9217 -1.0979 -0.7454 -0.9217 0.0899 0.05

A vs B at 15 -1.0328 -1.2230 -0.8426 -1.0328 0.0971 0.05

Contrast Estimate Results

L'Beta Chi-

Label Confidence Limits Square Pr > ChiSq

A vs B at 8 -1.2272 -0.4645 18.90 <.0001

A vs B at 9 -1.0003 -0.4085 21.77 <.0001

A vs B at 10 -1.1128 -0.7014 74.71 <.0001

A vs B at 11 -0.9507 -0.5072 41.50 <.0001

A vs B at 12 -0.9466 -0.5377 50.61 <.0001

A vs B at 13 -1.0607 -0.6702 75.46 <.0001

A vs B at 14 -1.0979 -0.7454 105.07 <.0001

A vs B at 15 -1.2230 -0.8426 113.22 <.0001

____________________________________________________________________________________________________

A = Blacks, B = Non-blacks

Table 1 GEE Model analysis of haematological parameters: Between and within group comparison

____________________________________________________________________________________________________

Label parameter=MONO(x10^8/L)

The GENMOD Procedure

Contrast Estimate Results

Mean Mean L'Beta Standard

Label Estimate Confidence Limits Estimate Error Alpha

A vs B at 16 -0.9795 -1.1492 -0.8097 -0.9795 0.0866 0.05

A vs B at 17 -0.7900 -1.0590 -0.5209 -0.7900 0.1373 0.05

A vs B at 18 -0.9745 -1.3033 -0.6458 -0.9745 0.1677 0.05

A vs B at 99 -0.8629 -0.9731 -0.7528 -0.8629 0.0562 0.05

Morning vs evening in A -0.2955 -0.5148 -0.0761 -0.2955 0.1119 0.05

Morning vs evening in B -0.3901 -0.4849 -0.2952 -0.3901 0.0484 0.05

Morning vs evening in X -0.3428 -0.4626 -0.2229 -0.3428 0.0611 0.05

Contrast Estimate Results

L'Beta Chi-

Label Confidence Limits Square Pr > ChiSq

A vs B at 16 -1.1492 -0.8097 127.90 <.0001

A vs B at 17 -1.0590 -0.5209 33.12 <.0001

A vs B at 18 -1.3033 -0.6458 33.75 <.0001

A vs B at 99 -0.9731 -0.7528 235.81 <.0001

Morning vs evening in A -0.5148 -0.0761 6.97 0.0083

Morning vs evening in B -0.4849 -0.2952 64.97 <.0001

Morning vs evening in X -0.4626 -0.2229 31.43 <.0001

treat*time Least Squares Means

Standard

treat time Estimate Error z Value Pr > |z| Alpha Lower Upper

A 0 4.5704 0.1852 24.67 <.0001 0.05 4.2073 4.9334

A 1 4.5721 0.1517 30.13 <.0001 0.05 4.2747 4.8695

A 2 4.3315 0.1050 41.25 <.0001 0.05 4.1257 4.5374

A 3 4.3401 0.1113 38.98 <.0001 0.05 4.1219 4.5583

A 4 4.3708 0.1049 41.67 <.0001 0.05 4.1652 4.5764

A 5 4.2700 0.09909 43.09 <.0001 0.05 4.0758 4.4642

A 6 4.3799 0.08987 48.74 <.0001 0.05 4.2037 4.5560

A 7 4.4763 0.09610 46.58 <.0001 0.05 4.2880 4.6647

A 8 4.5832 0.08785 52.17 <.0001 0.05 4.4111 4.7554

A 9 4.8417 0.1303 37.16 <.0001 0.05 4.5864 5.0971

A 10 4.9444 0.1458 33.90 <.0001 0.05 4.6586 5.2303

B 0 5.4162 0.08018 67.55 <.0001 0.05 5.2591 5.5734

B 1 5.2765 0.05915 89.21 <.0001 0.05 5.1606 5.3924

B 2 5.2386 0.05970 87.75 <.0001 0.05 5.1216 5.3557

B 3 5.0690 0.06105 83.03 <.0001 0.05 4.9494 5.1887

B 4 5.1130 0.05761 88.75 <.0001 0.05 5.0000 5.2259

B 5 5.1354 0.05597 91.75 <.0001 0.05 5.0257 5.2451

B 6 5.3015 0.05640 93.99 <.0001 0.05 5.1910 5.4121

B 7 5.5091 0.05781 95.30 <.0001 0.05 5.3958 5.6224

B 8 5.5627 0.05344 104.10 <.0001 0.05 5.4580 5.6674

B 9 5.6317 0.06782 83.04 <.0001 0.05 5.4987 5.7646

B 10 5.9190 0.09502 62.29 <.0001 0.05 5.7327 6.1052

____________________________________________________________________________________________________

A = Blacks, B = Non-blacks

Table 1 GEE Model analysis of haematological parameters: Between and within group comparison

____________________________________________________________________________________________________

Label parameter=NEUT(x10^9/L)

The GENMOD Procedure

Model Information

Data Set WORK.ENDPOINTS

Distribution Normal

Link Function Identity

Dependent Variable outcome

Number of Observations Read 12808

Number of Observations Used 12801

Missing Values 7

Class Level Information

Class Levels Values

study 75 RICH PHARM RP DFP RPC09003 RPC09004 RPC09008

RPC09029 RPC09051 RPC09052 RPC09054 RPC09068

RPC09069 RPC09070 RPC09071 RPC09079 RPC10009

RPC10021 RPC10027 RPC10035 RPC10045 RPC10050

RPC11002 RPC11003 RPC11035 RPC11050 RPC11061

RPC11067 RPC11069 RPC11089 ...

pat 7068 132 321 364 786 940 1213 2474 2535 2946 3040 3635

4026 4051 5656 5775 6017 6176 6646 6774 7094 8142

8560 8721 8805 8810 8924 9047 9051 9155 9624 10040

10308 10466 10563 10619 11604 11824 11980 12076

12431 12437 12712 13045 13575 13593 13946 14198

14404 ...

treat 2 A B

time 11 0 1 2 3 4 5 6 7 8 9 10

sex 2 F M

Fasted 2 N Y

Parameter Information

Parameter Effect treat time sex Fasted

Prm1 Intercept

Prm2 treat A

Prm3 treat B

Prm4 time 0

Prm5 time 1

Prm6 time 2

Prm7 time 3

Prm8 time 4

Prm9 time 5

Prm10 time 6

Prm11 time 7

Prm12 time 8

Prm13 time 9

Prm14 time 10

Prm15 treat*time A 0

Prm16 treat*time A 1

Prm17 treat*time A 2

Prm18 treat*time A 3

Prm19 treat*time A 4

Prm20 treat*time A 5

Prm21 treat*time A 6

Prm22 treat*time A 7

Prm23 treat*time A 8

Prm24 treat*time A 9

Prm25 treat*time A 10

Prm26 treat*time B 0

Prm27 treat*time B 1

____________________________________________________________________________________________________

A = Blacks, B = Non-blacks

Table 1 GEE Model analysis of haematological parameters: Between and within group comparison

____________________________________________________________________________________________________

Label parameter=NEUT(x10^9/L)

The GENMOD Procedure

Parameter Information

Parameter Effect treat time sex Fasted

Prm28 treat*time B 2

Prm29 treat*time B 3

Prm30 treat*time B 4

Prm31 treat*time B 5

Prm32 treat*time B 6

Prm33 treat*time B 7

Prm34 treat*time B 8

Prm35 treat*time B 9

Prm36 treat*time B 10

Prm37 age

Prm38 sex F

Prm39 sex M

Prm40 Fasted N

Prm41 Fasted Y

Algorithm converged.

GEE Model Information

Correlation Structure Exchangeable

Subject Effect pat (7071 levels)

Number of Clusters 7071

Clusters With Missing Values 7

Correlation Matrix Dimension 22

Maximum Cluster Size 22

Minimum Cluster Size 0

Algorithm converged.

Exchangeable Working

Correlation

Correlation 0.4689208473

GEE Fit Criteria

QIC 12814.7688

QICu 12826.0000

Analysis Of GEE Parameter Estimates

Empirical Standard Error Estimates

Standard 95% Confidence

Parameter Estimate Error Limits Z Pr > |Z|

Intercept 4.4292 0.0959 4.2413 4.6171 46.20 <.0001

treat A -1.4273 0.1340 -1.6899 -1.1647 -10.65 <.0001

treat B 0.0000 0.0000 0.0000 0.0000 . .

time 0 -1.2932 0.0954 -1.4802 -1.1061 -13.55 <.0001

time 1 -1.1107 0.0913 -1.2896 -0.9317 -12.17 <.0001

time 2 -0.8886 0.0909 -1.0667 -0.7104 -9.78 <.0001

time 3 -0.8901 0.0915 -1.0694 -0.7109 -9.73 <.0001

time 4 -0.7321 0.0906 -0.9097 -0.5545 -8.08 <.0001

time 5 -0.6186 0.0918 -0.7986 -0.4386 -6.74 <.0001

time 6 -0.3693 0.0955 -0.5565 -0.1820 -3.87 0.0001

time 7 -0.1494 0.0936 -0.3328 0.0340 -1.60 0.1104

____________________________________________________________________________________________________

A = Blacks, B = Non-blacks

Table 1 GEE Model analysis of haematological parameters: Between and within group comparison

____________________________________________________________________________________________________

Label parameter=NEUT(x10^9/L)

The GENMOD Procedure

Analysis Of GEE Parameter Estimates

Empirical Standard Error Estimates

Standard 95% Confidence

Parameter Estimate Error Limits Z Pr > |Z|

time 8 0.0069 0.0920 -0.1734 0.1872 0.08 0.9402

time 9 -0.0390 0.1001 -0.2353 0.1572 -0.39 0.6967

time 10 0.0000 0.0000 0.0000 0.0000 . .

treat*time A 0 0.3803 0.2092 -0.0297 0.7903 1.82 0.0691

treat*time A 1 0.3561 0.1493 0.0634 0.6487 2.38 0.0171

treat*time A 2 0.2657 0.1471 -0.0226 0.5540 1.81 0.0708

treat*time A 3 0.2967 0.1451 0.0124 0.5810 2.05 0.0408

treat*time A 4 0.2368 0.1477 -0.0527 0.5264 1.60 0.1088

treat*time A 5 0.2717 0.1507 -0.0238 0.5671 1.80 0.0715

treat*time A 6 -0.0114 0.1535 -0.3123 0.2896 -0.07 0.9409

treat*time A 7 -0.0295 0.1531 -0.3295 0.2705 -0.19 0.8470

treat*time A 8 -0.1871 0.1439 -0.4692 0.0949 -1.30 0.1934

treat*time A 9 -0.0952 0.1575 -0.4040 0.2135 -0.60 0.5456

treat*time A 10 0.0000 0.0000 0.0000 0.0000 . .

treat*time B 0 0.0000 0.0000 0.0000 0.0000 . .

treat*time B 1 0.0000 0.0000 0.0000 0.0000 . .

treat*time B 2 0.0000 0.0000 0.0000 0.0000 . .

treat*time B 3 0.0000 0.0000 0.0000 0.0000 . .

treat*time B 4 0.0000 0.0000 0.0000 0.0000 . .

treat*time B 5 0.0000 0.0000 0.0000 0.0000 . .

treat*time B 6 0.0000 0.0000 0.0000 0.0000 . .

treat*time B 7 0.0000 0.0000 0.0000 0.0000 . .

treat*time B 8 0.0000 0.0000 0.0000 0.0000 . .

treat*time B 9 0.0000 0.0000 0.0000 0.0000 . .

treat*time B 10 0.0000 0.0000 0.0000 0.0000 . .

age 0.0009 0.0014 -0.0018 0.0036 0.67 0.5032

sex F 0.1626 0.0322 0.0996 0.2257 5.06 <.0001

sex M 0.0000 0.0000 0.0000 0.0000 . .

Fasted N -0.0302 0.0646 -0.1568 0.0965 -0.47 0.6405

Fasted Y 0.0000 0.0000 0.0000 0.0000 . .

Contrast Estimate Results

Mean Mean L'Beta Standard

Label Estimate Confidence Limits Estimate Error Alpha

A vs B at 8 -1.0470 -1.3707 -0.7234 -1.0470 0.1651 0.05

A vs B at 9 -1.0713 -1.2326 -0.9099 -1.0713 0.0823 0.05

A vs B at 10 -1.1616 -1.3184 -1.0049 -1.1616 0.0800 0.05

A vs B at 11 -1.1307 -1.2933 -0.9681 -1.1307 0.0830 0.05

A vs B at 12 -1.1905 -1.3515 -1.0295 -1.1905 0.0821 0.05

A vs B at 13 -1.1557 -1.3205 -0.9909 -1.1557 0.0841 0.05

A vs B at 14 -1.4387 -1.6164 -1.2610 -1.4387 0.0907 0.05

A vs B at 15 -1.4569 -1.6231 -1.2906 -1.4569 0.0848 0.05

Contrast Estimate Results

L'Beta Chi-

Label Confidence Limits Square Pr > ChiSq

A vs B at 8 -1.3707 -0.7234 40.20 <.0001

A vs B at 9 -1.2326 -0.9099 169.34 <.0001

A vs B at 10 -1.3184 -1.0049 210.97 <.0001

A vs B at 11 -1.2933 -0.9681 185.78 <.0001

A vs B at 12 -1.3515 -1.0295 210.09 <.0001

A vs B at 13 -1.3205 -0.9909 188.87 <.0001

A vs B at 14 -1.6164 -1.2610 251.84 <.0001

A vs B at 15 -1.6231 -1.2906 295.09 <.0001

____________________________________________________________________________________________________

A = Blacks, B = Non-blacks

Table 1 GEE Model analysis of haematological parameters: Between and within group comparison

____________________________________________________________________________________________________

Label parameter=NEUT(x10^9/L)

The GENMOD Procedure

Contrast Estimate Results

Mean Mean L'Beta Standard

Label Estimate Confidence Limits Estimate Error Alpha

A vs B at 16 -1.6145 -1.7634 -1.4656 -1.6145 0.0760 0.05

A vs B at 17 -1.5225 -1.7088 -1.3363 -1.5225 0.0950 0.05

A vs B at 18 -1.4273 -1.6899 -1.1647 -1.4273 0.1340 0.05

A vs B at 99 -1.2924 -1.3760 -1.2088 -1.2924 0.0427 0.05

Morning vs evening in A -0.6520 -0.8016 -0.5025 -0.6520 0.0763 0.05

Morning vs evening in B -1.0759 -1.1581 -0.9937 -1.0759 0.0419 0.05

Morning vs evening in X -0.8640 -0.9497 -0.7782 -0.8640 0.0437 0.05

Contrast Estimate Results

L'Beta Chi-

Label Confidence Limits Square Pr > ChiSq

A vs B at 16 -1.7634 -1.4656 451.59 <.0001

A vs B at 17 -1.7088 -1.3363 256.68 <.0001

A vs B at 18 -1.6899 -1.1647 113.49 <.0001

A vs B at 99 -1.3760 -1.2088 918.01 <.0001

Morning vs evening in A -0.8016 -0.5025 73.02 <.0001

Morning vs evening in B -1.1581 -0.9937 658.31 <.0001

Morning vs evening in X -0.9497 -0.7782 390.25 <.0001

treat*time Least Squares Means

Standard

treat time Estimate Error z Value Pr > |z| Alpha Lower Upper

A 0 2.1844 0.1606 13.60 <.0001 0.05 1.8697 2.4991

A 1 2.3427 0.08118 28.86 <.0001 0.05 2.1836 2.5018

A 2 2.4744 0.07939 31.17 <.0001 0.05 2.3188 2.6300

A 3 2.5038 0.08200 30.53 <.0001 0.05 2.3431 2.6645

A 4 2.6020 0.08170 31.85 <.0001 0.05 2.4419 2.7622

A 5 2.7503 0.08172 33.66 <.0001 0.05 2.5902 2.9105

A 6 2.7166 0.08667 31.34 <.0001 0.05 2.5467 2.8865

A 7 2.9184 0.08159 35.77 <.0001 0.05 2.7584 3.0783

A 8 2.9170 0.07171 40.68 <.0001 0.05 2.7765 3.0576

A 9 2.9630 0.07994 37.07 <.0001 0.05 2.8064 3.1197

A 10 3.0973 0.1057 29.31 <.0001 0.05 2.8902 3.3044

B 0 3.2314 0.05757 56.13 <.0001 0.05 3.1186 3.3443

B 1 3.4140 0.04791 71.26 <.0001 0.05 3.3201 3.5079

B 2 3.6360 0.04834 75.22 <.0001 0.05 3.5413 3.7308

B 3 3.6345 0.04708 77.20 <.0001 0.05 3.5422 3.7267

B 4 3.7925 0.04774 79.45 <.0001 0.05 3.6989 3.8861

B 5 3.9060 0.04777 81.78 <.0001 0.05 3.8124 3.9996

B 6 4.1553 0.05313 78.22 <.0001 0.05 4.0512 4.2594

B 7 4.3752 0.04910 89.11 <.0001 0.05 4.2790 4.4715

B 8 4.5315 0.04867 93.11 <.0001 0.05 4.4361 4.6269

B 9 4.4856 0.06131 73.16 <.0001 0.05 4.3654 4.6058

B 10 4.5246 0.08908 50.79 <.0001 0.05 4.3500 4.6992

____________________________________________________________________________________________________

A = Blacks, B = Non-blacks

Table 1 GEE Model analysis of haematological parameters: Between and within group comparison

____________________________________________________________________________________________________

Label parameter=WBC(x10^9/L)

The GENMOD Procedure

Model Information

Data Set WORK.ENDPOINTS

Distribution Normal

Link Function Identity

Dependent Variable outcome

Number of Observations Read 12950

Number of Observations Used 12943

Missing Values 7

Class Level Information

Class Levels Values

study 76 RICH PHARM RP DFP RPC09003 RPC09004 RPC09008

RPC09029 RPC09051 RPC09052 RPC09054 RPC09068

RPC09069 RPC09070 RPC09071 RPC09079 RPC10009

RPC10021 RPC10027 RPC10035 RPC10045 RPC10050

RPC11002 RPC11003 RPC11035 RPC11050 RPC11061

RPC11067 RPC11069 RPC11089 ...

pat 7070 132 321 364 786 940 1213 2474 2535 2946 3040 3635

4026 4051 5656 5775 6017 6176 6646 6774 7094 8142

8560 8721 8805 8810 8924 9047 9051 9155 9624 10040

10308 10466 10563 10619 11604 11824 11980 12076

12431 12437 12712 13045 13575 13593 13946 14198

14404 ...

treat 2 A B

time 11 0 1 2 3 4 5 6 7 8 9 10

sex 2 F M

Fasted 2 N Y

Parameter Information

Parameter Effect treat time sex Fasted

Prm1 Intercept

Prm2 treat A

Prm3 treat B

Prm4 time 0

Prm5 time 1

Prm6 time 2

Prm7 time 3

Prm8 time 4

Prm9 time 5

Prm10 time 6

Prm11 time 7

Prm12 time 8

Prm13 time 9

Prm14 time 10

Prm15 treat*time A 0

Prm16 treat*time A 1

Prm17 treat*time A 2

Prm18 treat*time A 3

Prm19 treat*time A 4

Prm20 treat*time A 5

Prm21 treat*time A 6

Prm22 treat*time A 7

Prm23 treat*time A 8

Prm24 treat*time A 9

Prm25 treat*time A 10

Prm26 treat*time B 0

Prm27 treat*time B 1

____________________________________________________________________________________________________

A = Blacks, B = Non-blacks

Table 1 GEE Model analysis of haematological parameters: Between and within group comparison

____________________________________________________________________________________________________

Label parameter=WBC(x10^9/L)

The GENMOD Procedure

Parameter Information

Parameter Effect treat time sex Fasted

Prm28 treat*time B 2

Prm29 treat*time B 3

Prm30 treat*time B 4

Prm31 treat*time B 5

Prm32 treat*time B 6

Prm33 treat*time B 7

Prm34 treat*time B 8

Prm35 treat*time B 9

Prm36 treat*time B 10

Prm37 age

Prm38 sex F

Prm39 sex M

Prm40 Fasted N

Prm41 Fasted Y

Algorithm converged.

GEE Model Information

Correlation Structure Exchangeable

Subject Effect pat (7073 levels)

Number of Clusters 7073

Clusters With Missing Values 7

Correlation Matrix Dimension 22

Maximum Cluster Size 22

Minimum Cluster Size 0

Algorithm converged.

Exchangeable Working

Correlation

Correlation 0.5518687976

GEE Fit Criteria

QIC 12955.2684

QICu 12968.0000

Analysis Of GEE Parameter Estimates

Empirical Standard Error Estimates

Standard 95% Confidence

Parameter Estimate Error Limits Z Pr > |Z|

Intercept 7.3651 0.1085 7.1524 7.5778 67.87 <.0001

treat A -1.4770 0.1554 -1.7816 -1.1724 -9.51 <.0001

treat B 0.0000 0.0000 0.0000 0.0000 . .

time 0 -1.5614 0.1102 -1.7773 -1.3455 -14.17 <.0001

time 1 -1.5129 0.1020 -1.7128 -1.3129 -14.83 <.0001

time 2 -1.3205 0.1009 -1.5182 -1.1229 -13.09 <.0001

time 3 -1.4181 0.1017 -1.6173 -1.2189 -13.95 <.0001

time 4 -1.1902 0.1006 -1.3874 -0.9931 -11.83 <.0001

time 5 -1.0529 0.1015 -1.2518 -0.8539 -10.37 <.0001

time 6 -0.6921 0.1054 -0.8986 -0.4856 -6.57 <.0001

time 7 -0.4023 0.1040 -0.6061 -0.1984 -3.87 0.0001

____________________________________________________________________________________________________

A = Blacks, B = Non-blacks

Table 1 GEE Model analysis of haematological parameters: Between and within group comparison

____________________________________________________________________________________________________

Label parameter=WBC(x10^9/L)

The GENMOD Procedure

Analysis Of GEE Parameter Estimates

Empirical Standard Error Estimates

Standard 95% Confidence

Parameter Estimate Error Limits Z Pr > |Z|

time 8 -0.1802 0.1013 -0.3788 0.0184 -1.78 0.0753

time 9 -0.1641 0.1106 -0.3809 0.0528 -1.48 0.1381

time 10 0.0000 0.0000 0.0000 0.0000 . .

treat*time A 0 0.4247 0.2415 -0.0486 0.8980 1.76 0.0786

treat*time A 1 0.3697 0.1748 0.0272 0.7122 2.12 0.0344

treat*time A 2 0.1858 0.1731 -0.1534 0.5250 1.07 0.2831

treat*time A 3 0.3988 0.1739 0.0580 0.7396 2.29 0.0218

treat*time A 4 0.2144 0.1718 -0.1222 0.5511 1.25 0.2119

treat*time A 5 0.2115 0.1753 -0.1322 0.5551 1.21 0.2278

treat*time A 6 -0.0478 0.1778 -0.3963 0.3006 -0.27 0.7879

treat*time A 7 -0.0850 0.1772 -0.4323 0.2623 -0.48 0.6314

treat*time A 8 -0.2512 0.1682 -0.5809 0.0784 -1.49 0.1352

treat*time A 9 -0.0651 0.1787 -0.4154 0.2853 -0.36 0.7158

treat*time A 10 0.0000 0.0000 0.0000 0.0000 . .

treat*time B 0 0.0000 0.0000 0.0000 0.0000 . .

treat*time B 1 0.0000 0.0000 0.0000 0.0000 . .

treat*time B 2 0.0000 0.0000 0.0000 0.0000 . .

treat*time B 3 0.0000 0.0000 0.0000 0.0000 . .

treat*time B 4 0.0000 0.0000 0.0000 0.0000 . .

treat*time B 5 0.0000 0.0000 0.0000 0.0000 . .

treat*time B 6 0.0000 0.0000 0.0000 0.0000 . .

treat*time B 7 0.0000 0.0000 0.0000 0.0000 . .

treat*time B 8 0.0000 0.0000 0.0000 0.0000 . .

treat*time B 9 0.0000 0.0000 0.0000 0.0000 . .

treat*time B 10 0.0000 0.0000 0.0000 0.0000 . .

age 0.0043 0.0018 0.0008 0.0077 2.44 0.0145

sex F 0.2297 0.0400 0.1513 0.3081 5.74 <.0001

sex M 0.0000 0.0000 0.0000 0.0000 . .

Fasted N 0.0324 0.0661 -0.0971 0.1620 0.49 0.6238

Fasted Y 0.0000 0.0000 0.0000 0.0000 . .

Contrast Estimate Results

Mean Mean L'Beta Standard

Label Estimate Confidence Limits Estimate Error Alpha

A vs B at 8 -1.0523 -1.4308 -0.6737 -1.0523 0.1931 0.05

A vs B at 9 -1.1073 -1.3045 -0.9101 -1.1073 0.1006 0.05

A vs B at 10 -1.2912 -1.4840 -1.0984 -1.2912 0.0984 0.05

A vs B at 11 -1.0782 -1.2912 -0.8652 -1.0782 0.1087 0.05

A vs B at 12 -1.2626 -1.4562 -1.0689 -1.2626 0.0988 0.05

A vs B at 13 -1.2655 -1.4602 -1.0709 -1.2655 0.0993 0.05

A vs B at 14 -1.5248 -1.7308 -1.3188 -1.5248 0.1051 0.05

A vs B at 15 -1.5620 -1.7600 -1.3640 -1.5620 0.1010 0.05

Contrast Estimate Results

L'Beta Chi-

Label Confidence Limits Square Pr > ChiSq

A vs B at 8 -1.4308 -0.6737 29.68 <.0001

A vs B at 9 -1.3045 -0.9101 121.10 <.0001

A vs B at 10 -1.4840 -1.0984 172.27 <.0001

A vs B at 11 -1.2912 -0.8652 98.40 <.0001

A vs B at 12 -1.4562 -1.0689 163.30 <.0001

A vs B at 13 -1.4602 -1.0709 162.32 <.0001

A vs B at 14 -1.7308 -1.3188 210.47 <.0001

A vs B at 15 -1.7600 -1.3640 239.10 <.0001

____________________________________________________________________________________________________

A = Blacks, B = Non-blacks

Table 1 GEE Model analysis of haematological parameters: Between and within group comparison

____________________________________________________________________________________________________

Label parameter=WBC(x10^9/L)

The GENMOD Procedure

Contrast Estimate Results

Mean Mean L'Beta Standard

Label Estimate Confidence Limits Estimate Error Alpha

A vs B at 16 -1.7282 -1.9070 -1.5495 -1.7282 0.0912 0.05

A vs B at 17 -1.5421 -1.7620 -1.3222 -1.5421 0.1122 0.05

A vs B at 18 -1.4770 -1.7816 -1.1724 -1.4770 0.1554 0.05

A vs B at 99 -1.3537 -1.4569 -1.2506 -1.3537 0.0526 0.05

Morning vs evening in A -0.9088 -1.0846 -0.7330 -0.9088 0.0897 0.05

Morning vs evening in B -1.3367 -1.4326 -1.2407 -1.3367 0.0489 0.05

Morning vs evening in X -1.1227 -1.2232 -1.0222 -1.1227 0.0513 0.05

Contrast Estimate Results

L'Beta Chi-

Label Confidence Limits Square Pr > ChiSq

A vs B at 16 -1.9070 -1.5495 359.04 <.0001

A vs B at 17 -1.7620 -1.3222 188.94 <.0001

A vs B at 18 -1.7816 -1.1724 90.35 <.0001

A vs B at 99 -1.4569 -1.2506 661.78 <.0001

Morning vs evening in A -1.0846 -0.7330 102.66 <.0001

Morning vs evening in B -1.4326 -1.2407 745.90 <.0001

Morning vs evening in X -1.2232 -1.0222 479.63 <.0001

treat*time Least Squares Means

Standard

treat time Estimate Error z Value Pr > |z| Alpha Lower Upper

A 0 5.0175 0.1843 27.22 <.0001 0.05 4.6562 5.3787

A 1 5.0110 0.09680 51.77 <.0001 0.05 4.8212 5.2007

A 2 5.0193 0.09555 52.53 <.0001 0.05 4.8321 5.2066

A 3 5.1348 0.1059 48.47 <.0001 0.05 4.9271 5.3424

A 4 5.1783 0.09650 53.66 <.0001 0.05 4.9892 5.3675

A 5 5.3127 0.09567 55.53 <.0001 0.05 5.1252 5.5002

A 6 5.4142 0.09957 54.37 <.0001 0.05 5.2191 5.6094

A 7 5.6668 0.09582 59.14 <.0001 0.05 5.4790 5.8546

A 8 5.7226 0.08543 66.99 <.0001 0.05 5.5552 5.8901

A 9 5.9250 0.09531 62.16 <.0001 0.05 5.7382 6.1118

A 10 6.1541 0.1255 49.03 <.0001 0.05 5.9081 6.4001

B 0 6.0697 0.07186 84.46 <.0001 0.05 5.9289 6.2106

B 1 6.1182 0.05339 114.59 <.0001 0.05 6.0136 6.2229

B 2 6.3106 0.05382 117.25 <.0001 0.05 6.2051 6.4160

B 3 6.2130 0.05314 116.92 <.0001 0.05 6.1088 6.3171

B 4 6.4409 0.05259 122.47 <.0001 0.05 6.3378 6.5440

B 5 6.5782 0.05281 124.56 <.0001 0.05 6.4747 6.6817

B 6 6.9390 0.05786 119.93 <.0001 0.05 6.8256 7.0524

B 7 7.2288 0.05566 129.88 <.0001 0.05 7.1197 7.3379

B 8 7.4509 0.05439 136.98 <.0001 0.05 7.3443 7.5575

B 9 7.4670 0.06817 109.53 <.0001 0.05 7.3334 7.6007

B 10 7.6311 0.09772 78.09 <.0001 0.05 7.4396 7.8226

____________________________________________________________________________________________________

A = Blacks, B = Non-blacks
